# Reconsidering priorities for forest conservation when considering the threats of mining and armed conflict

**DOI:** 10.1007/s13280-022-01724-0

**Published:** 2022-04-10

**Authors:** Brooke A. Williams, Hedley S. Grantham, James E. M. Watson, Aurélie C. Shapiro, Andrew J. Plumptre, Samuel Ayebare, Elizabeth Goldman, Ayesha I. T. Tulloch

**Affiliations:** 1 School of Earth and Environmental Sciences, The University of Queensland, St Lucia, QLD 4072 Australia; 2 Centre for Biodiversity and Conservation Science, The University of Queensland, St Lucia, QLD 4072 Australia; 3 Global Conservation Program, Wildlife Conservation Society, Bronx, NY 10460-1068 USA; 4 Geography Department, Humboldt-Universität-zu-Berlin, Berlin, Germany; 5 Food and Agriculture Organization of the United Nations, Rome, Italy; 6 Key Biodiversity Areas Secretariat, c/o BirdLife International, David Attenborough Building, Pembroke Street, Cambridge, UK; 7 Conservation Science Group, Zoology Department, Cambridge University, Pembroke St, Cambridge, UK; 8 Albertine Rift Program, Wildlife Conservation Society, PO Box 7487, Kampala, Uganda; 9 World Resources Institute, Washington, DC 20002 USA; 10 School of Life and Environmental Sciences, The University of Sydney, Camperdown, NSW 2006 Australia; 11 School of Biology and Environmental Science, Queensland University of Technology, Brisbane, QLD 4000 Australia

**Keywords:** Anthropogenic threatening process, Deforestation, Democratic Republic of the Congo, Spatial conservation planning, Species distribution model, Zonation

## Abstract

Many threats to biodiversity can be predicted and are well mapped but others are uncertain in their extent, impact on biodiversity, and ability for conservation efforts to address, making them more difficult to account for in spatial conservation planning efforts, and as a result, they are often ignored. Here, we use a spatial prioritisation analysis to evaluate the consequences of considering only relatively well-mapped threats to biodiversity and compare this with planning scenarios that also account for more uncertain threats (in this case mining and armed conflict) under different management strategies. We evaluate three management strategies to address these more uncertain threats: 1. to ignore them; 2. avoid them; or 3. specifically target actions towards them, first individually and then simultaneously to assess the impact of their inclusion in spatial prioritisations. We apply our approach to the eastern Democratic Republic of the Congo (DRC) and identify priority areas for conserving biodiversity and carbon sequestration services. We found that a strategy that avoids addressing threats of mining and armed conflict more often misses important opportunities for biodiversity conservation, compared to a strategy that targets action towards areas under threat (assuming a biodiversity benefit is possible). We found that considering mining and armed conflict threats to biodiversity independently rather than simultaneously results in 13 800–14 800 km^2^ and 15 700–25 100 km^2^ of potential missed conservation opportunities when undertaking threat-avoiding and threat-targeting management strategies, respectively. Our analysis emphasises the importance of considering all threats that can be mapped in spatial conservation prioritisation.

**Supplementary Information:**

The online version contains supplementary material available at 10.1007/s13280-022-01724-0.

## Introduction

With the increase in Earth observation technologies (Buchanan et al. 2009), many threats (that is, activities or processes that have caused, are causing, or may cause the destruction, degradation, and/or impairment of biodiversity targets (e.g. unsustainable fishing or logging) (Salafsky et al. 2008)) to biodiversity are now more easily detectable across landscapes than previously (Tulloch et al. 2015a, b; Joppa et al. 2016). In forested ecosystems, some relatively well-mapped threats include permanent land-use change related to industrial forestry activities (Fagan et al. 2018), agriculture (Buchhorn et al. 2020), wildfire (Artés et al. 2019) and urbanisation (Small et al. 2011; Zhou et al. 2015). But these remote sensing methods cannot detect all threatening processes and some threats remain highly uncertain in terms of extent and impact (a measure of whether the changes in the state variables have a negative or positive effect on individuals, society and/or environmental resources (Harrington et al. 2010)) (e.g. difficult to detect invasive species, small-scale resource extraction activities or species-specific impacts of roads). Problematically, these threats are not always directly correlated with other predictable pressures (Joppa et al. 2016). Therefore, the impacts of these more uncertain threats are often difficult to map or manage and can result in both short- and long-term environmental damage (Gaynor et al. 2016; Spira et al. 2017; Baker et al. 2018).

Although threat data are increasingly used when planning conservation actions (Tulloch et al. 2016), some threats are routinely overlooked in large-scale regional planning. This may be because they are difficult to study and map due to unpredictable drivers (such as those driven by socio-political factors), are remote in nature and difficult to access or because the data simply do not exist on their extents (Murray et al. 2014; Stephanson and Mascia 2014; Gaynor et al. 2016; Reid et al. 2019). Failing to account for any type of threat can lead to the failure of conservation actions or missed conservation opportunities, as threat data have been shown to drive the outcomes of spatial prioritisations (Evans et al. 2015; Joppa et al. 2016; Kujala et al. 2018). One of the reasons is that many threats spatially co-occur with areas of high value for biodiversity (Allan et al. 2019). For example, the areas valuable to human communities for resources (and therefore likely to be impacted by anthropogenic threats such as clearing for urban development, farming and hunting) are generally those with high productivity and associated species diversities and abundances (Cardiff and Andriamanalina 2007; Hirons 2011; Durán et al. 2013).

Across the Democratic Republic of the Congo (DRC), well-mapped threats including population growth, subsistence agriculture and urban development (Ernst et al. 2013) are inter-connected with the threats of artisanal and small-scale mining (ASM; informal mining activities carried out using low technology or with minimal machinery), and conflicts due to human warfare (engagement in or the activities involved in war or violent conflict) (Butsic et al. 2015). While the locations of artisanal and small-scale mines are increasingly well known (thanks to fine-scale satellite mapping of human alterations to landscapes and bottom-up mapping surveys), the impacts of ASM on biodiversity are difficult to account for but are numerous (IGF 2017). In the DRC, as in many other developing nations of Africa (Banchirigah 2006), artisanal mines draw local people to remote locations in the forest that are often inaccessible to law enforcement or are guarded by armed groups or militia, especially if they are illegal (Spira et al. 2017). Environmental practices tend to be poor as ASM relies on a mostly unskilled workforce and are often completely unregulated (IGF 2017). Mine impacts are local (site-level clearing for the mine) and also diffuse across the landscape through pollution of air and water resources by dust, heavy metals and fine particles, and with the increase of roads and human populations that are associated with mining activities, selective wood collection, and bushmeat hunting at considerable distances to sustain the miners and their families (Spira et al. 2017). ASM in the DRC has evolved with the armed conflict of the region. Frequent displacement, the fear of violence, inability to travel safely and the disintegration of agricultural markets have all contributed to the decline of previous forms of income generation. ASM now provides a more viable employment opportunity than farming, supporting around 14–16% of the DRC’s population (Kelly 2014; Spira et al. 2017).

The DRC has been affected by instability since the beginning of the 1990s and by all-out civil war from 1996 to 2003. Although the Second Congo War officially ended in 2003, conflict continues within the eastern part of the country. Displaced civilians are often forced to flee the unrest, and armed groups and soldiers are drawn into remote areas of the forest, both of which must rely heavily on forest resources to survive (Draulans and Van Krunkelsven 2002). While some studies report benefits to biodiversity (for example, through exclusion zones; Hammill et al. 2016), a breakdown of governance associated with this conflict has also facilitated activities such as poaching of animals, live animal trade, unsustainable logging and deforestation and also made managing protected areas difficult in many parts of the conflict-affected areas of the DRC (Draulans and Van Krunkelsven 2002).

The DRC holds the second largest extent of tropical forest after Brazil (Xu et al. 2017) and is therefore globally significant for tropical biodiversity conservation (Mwinyihali and Hart 2001) and carbon sequestration and storage (Xu et al. 2017). The presence of conflict and mining in the landscape is a significant concern and makes planning that accounts for these threats a challenge for conservation practitioners in the region (Tulloch and Grantham 2019). Previous analyses in the DRC have identified priorities for protection that avoided well-studied threats such as agriculture, hunting access and habitat degradation (Rondinini et al. 2006; Nackoney and Williams 2013; Grantham et al. 2020a, b). It is possible that selecting to avoid certain well-mapped threats may have resulted in priorities driven by information about these threats alone (Hammill et al. 2016), rather than by the ultimate objectives of maintaining biodiversity and carbon sequestration services (Tulloch et al. 2015a, b). A study from the Albertine Rift, partially included in the eastern DRC, found that through strategic planning, most species could be preserved outside existing mining concessions. However, there were some areas where conservation needed to take place within planned mining concessions to achieve the conservation targets (Plumptre et al. 2020). In other words, avoiding mining concessions missed important conservation opportunities.

An alternative approach to avoiding threats is to address them directly in conservation planning by incorporating specific objectives related to biodiversity retention, restoration, and recovery. Such objectives might lead to prioritisation of an action to help mitigate an ongoing threat (if it is possible to do so), or if a threat is not permanent, to improve the condition of a priority area once the threat has been alleviated or is no longer operating in the case of threats limited by environmental resources (Ando et al. 2018; Liang et al. 2018). Targeting action towards threats assumes that locations affected by a given threat may be recovered and may have higher value for biodiversity than those unaffected by that threat (Tulloch et al. 2019).

When locations with more uncertain threats are avoided, there is potential to miss actions or locations where large gains can be made towards biodiversity conservation. At the other extreme, if conservation action is targeted only towards these threatened locations, an action or location may be selected that has such low probability of success that management will eventually fail (Wilson et al. 2005a, b). For example, implementing a conservation action in a location targeted by ASM without support from local stakeholders will have a high chance of failure (Chan et al. 2007). Additionally, it is often impossible to meet conservation objectives by completely avoiding these more uncertain threats, as many species now exist only in threatened, fragmented ecosystems (Witt and Hammill 2018). Any decision therefore requires considering trade-offs, by evaluating and providing a variety of spatially explicit solutions and their potential outcomes to conservation practitioners.

Here, we identify priority areas for conservation action that support biodiversity (species and ecosystems) outcomes and an essential ecosystem service relating to mitigating climate change (carbon storage), and are currently impacted by relatively well-mapped (direct habitat loss, proximity to human settlements, population density and access) and more uncertain threats (artisanal mining and conflict), using the case study of the eastern DRC. We utilise the decision support tool Zonation (Moilanen et al. 2014) to compare three common management strategies specifically targeted at addressing mining and conflict impacts: 1. to ignore them (hereafter a threat-ignorant strategy); 2. avoid them (threat-avoiding strategy); or 3. specifically account for them and target actions towards them (threat-accounting strategy). We explore scenarios that include future impacts to biodiversity from artisanal mining and conflict individually and then simultaneously to assess the impact of how they are included in spatial prioritisation. Based on the spatial priorities identified within each scenario, we quantify the implications of each scenario and management strategy (to ignore, avoid, or account for threats) for biodiversity known to be at risk. The results from this study will help inform efforts trying to understand the consequences for conservation priorities when different threats are captured or ignored. At a regional scale, these analyses can help inform conservation decisions across the eastern DRC and the broader methodological framework can be applied to any landscape where both well-mapped and more uncertain threats exist.

## Materials and methods

### Study region

**Fig. 1:**
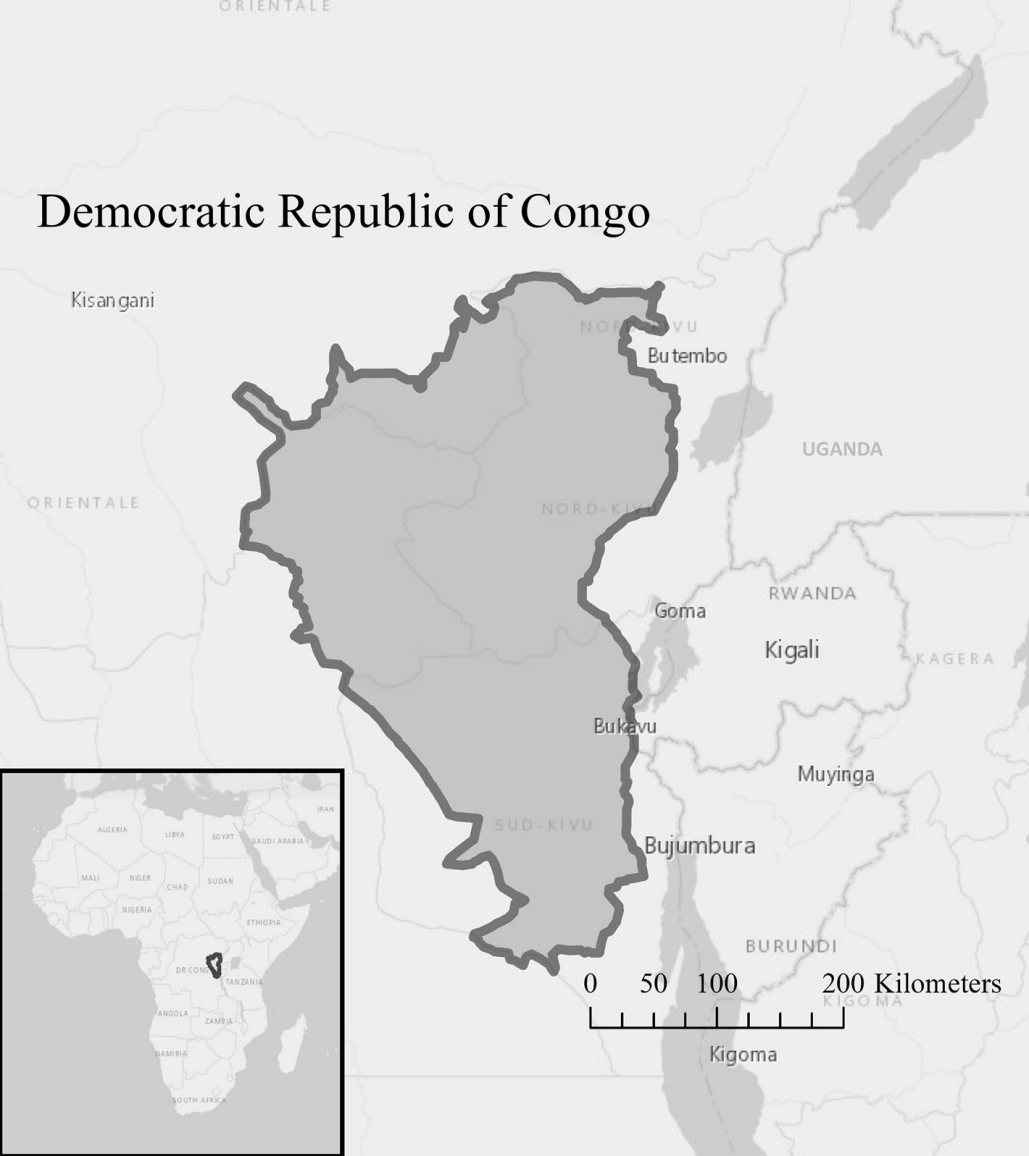
The Maiko–Tayna–Kahuzi-Biega Landscape, the Democratic Republic of the Congo

The Maiko-Tayna-Kahuzi-Biega landscape within the eastern DRC (Fig. 1) is one of nine landscapes identified by CARPE (Central Africa Regional Program for the Environment) as a priority area for conservation action by the international community (USAID 2015). The landscape boundary covers 106 096 km^2^, encompassing the Albertine Rift mountains to the east, down to the lowlands near the Congo River in the west (USAID 2015). It contains numerous important species such as the endangered eastern chimpanzee (*Pan troglodytes schweinfurthii*) and critically endangered Grauer’s gorilla (*Gorilla beringei graueri*) (Maldonado et al. 2012) and contains two globally recognised endemic bird areas (Stattersfield 1998). The region represents one of the largest expanses of intact forest in Central Africa (Grantham et al. 2020a, b), making it an important area for carbon sequestration services (USAID 2015).

### Overview

**Fig. 2:**
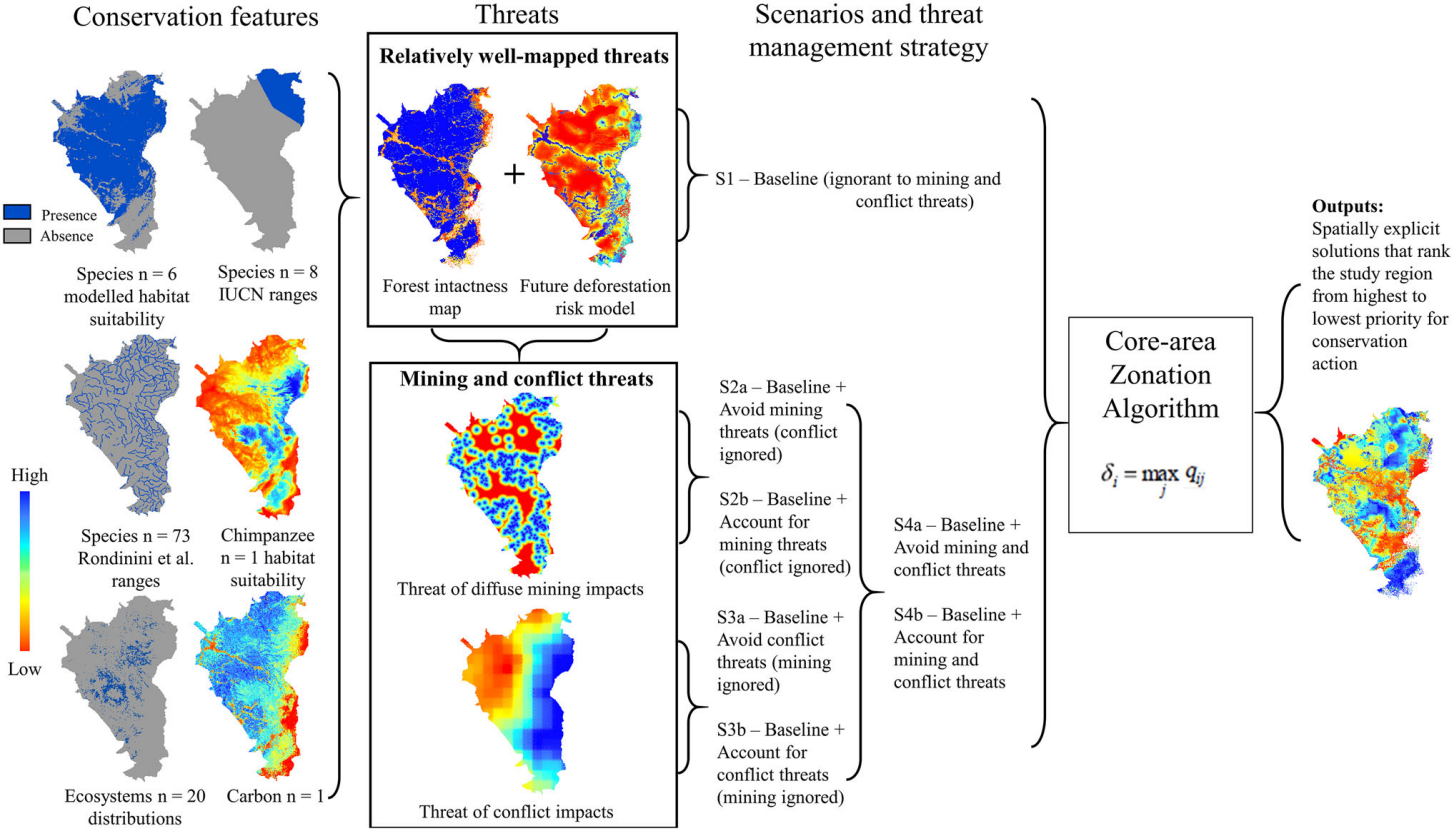
Methodological flow diagram of the input data (conservation features and threats), scenarios (S1, S2 and S3), and mining and conflict threat management strategies (threat-ignorant, threat-avoiding (a) or threat-accounting (b)) used to develop exact spatially explicit ranked solutions solved using Zonation Spatial Conservation Planning Software (Moilanen et al. 2014). Datasets were continuous rasters for chimpanzee suitability and carbon, and binary rasters for modelled species habitat suitability (*n* = 6), IUCN species ranges (*n* = 8), Rondinini et al. 2011 species ranges (*n* = 73), ecosystems (*n* = 20) and continuous rasters for all threats. The images under the conservation features column are an example of each data type where there are multiple features within each category (species and ecosystem categories), and *n* represents how many features there are in each category. For a full list of included features, see Appendix A

We used spatial prioritisation to achieve an objective of maximising representation of all conservation features (species, ecosystems and above- and belowground carbon) within the highest ranked cells while accounting for different combinations of well-mapped and uncertain threats. We focussed on the well-mapped threats of direct habitat loss, proximity to human settlements, population density, and access, and uncertain threats of artisanal mining and conflict, using different strategies (threat-ignorant, threat-avoiding, threat-accounting (sensu Hammill et al. 2016)) for considering future threats to a conservation feature at a site (Fig. 2). For the latter two threats, although they can occur at relatively small scales, their indirect impacts are diffuse across the landscape (Ingram et al. 2011; Gaynor et al. 2016). We explored three strategies for accounting for these two uncertain threats in spatial prioritisation (see “Scenarios” section): 1. ignoring threats of mining and conflict and considering only future well-mapped threats (a threat-ignorant strategy); 2. avoiding mining and conflict threats, assuming that threatened areas provide no biodiversity benefit and are avoided in favour of areas with higher biodiversity benefit (a threat-avoiding strategy); and 3. accepting risk in threatened locations by allowing the prioritisation to target action towards threats, which assumes that although a threat may be present, there is still opportunity for a successful conservation outcome (a threat-accounting strategy). We included the threats from conflict and mining within each scenario first individually, then simultaneously (Fig. 2).

### Data

#### Conservation features

Mammals make up the bulk of hunted species (Fa and Brown 2009; Ripple et al. 2016); therefore, we included the 88 mammal species in the region with hunting listed as a threat by the IUCN using the redlist package in R (Chamberlain 2017; IUCN 2020a). These species were from the orders *Afrosoricida, Carnivora, Certartiodactyla, Chiroptera, Eulipotyphla, Hyracoidea, Macroscelidea, Pholidota, Primates, Proboscidea, Rodentia* and *Tubulidentata*. We used either species distribution models (SDMs) or range maps to represent the distribution of each species in the area, with the choice of method determined by existing data availability. For one species, chimpanzees (*Pan troglodytes*), we used a high-resolution (approximately 28 m^2^) SDM, specifically a hSDM zero-inflated binomial (ZIB) model, that had previously been developed using species occupancy estimates to estimate probability of occurrence (in the study region, the range of probability is between 0 and 0.97) (Plumptre et al. 2015). For six other species (*Crocuta crocuta, Panthera leo, Hippopotamus amphibious, Okapia johnstoni, Gorilla beringei* and *Loxodonta africana*), we used 1 km^2^ binary habitat suitability raster layers (Plumptre et al. 2016). For the remaining species where models were not available and occurrence data were rare, we used species range maps from Rondinini et al. (2011) (rasters) which are habitat suitability models based on IUCN ranges and species habitat relationships (*n* = 73). For eight species not available in Rondinini et al., we used range maps (polygons) from the International Union for Conservation of Nature (IUCN) (IUCN 2020b). All species maps were converted to rasters (if they were not already) with a 1 km^2^ resolution.

The distributions of 20 forest ecosystems, as defined by Shapiro et al. 2021, were also included as conservation features to ensure the persistence of a diverse range of natural habitats for species assemblages, ecological process and provisioning services (Pressey et al. 2003; Loreau et al. 2006). Additionally, it is increasingly recognised that forests play a key role in mitigating climate change through carbon storage and sequestration (Watson et al. 2018). We therefore included above- and belowground carbon density as a feature, defined by Xu et al. (2017). The model was created using an airborne LiDAR inventory of more than 432 000 ha of forests based on a designed probability sampling methodology. The LiDAR mean top canopy height measurements were trained to develop an unbiased carbon estimator by using 92 one ha ground plots distributed across key forest types in the DRC. LiDAR samples provided estimates of mean and uncertainty of aboveground carbon density at provincial scales and were combined with optical and radar satellite imagery in a machine learning algorithm to map forest height and carbon density over the entire country (Xu et al. 2017). In total, 109 individual feature layers were included, see Appendix A for complete list of included features.

#### Threats

We use a variety of threat maps in our analysis and assume that the presence, likelihood of a threat occurring, or distance to a threat correlate with its relative impact on biodiversity (Tulloch et al. 2015a, b). To represent current and future well-mapped threats, we used a forest intactness map from Grantham et al. (2020a, b) which includes the current mappable threats of direct forest loss and fragmentation (remotely sensed), proximity to human settlements, population density and accessibility and combined it with a map depicting the results of a future deforestation risk model which is driven by predictable biophysical and anthropogenic factors of forest loss (Goldman et al. 2017). These two raster layers were linearly rescaled to values between 0 and 1 and then multiplied such that a value of 1 represents areas that have almost no threats, are the most “intact” in terms of overall forest cover and fragmentation and are least threatened by future anthropogenic activities.

For conflict, we used a previously published logistic model developed by Hammill et al. (2016) that predicts conflict risk across the study region (within each planning unit) based on conflict history. The statistical model represents the conflict risk using local history of conflicts and is parameterised by data obtained from the Institute for Economics and Peace (Global Peace Index 2016), from Hegre et al. (2013), and for previous conflict incidents, from the Armed Conflict Location and Event Dataset (ACLED; Raleigh et al. 2010). To calibrate the model, Hammill et al. (2016) used the data from 10 years of the ACLED database (1999–2008) to ‘predict’ risk in the subsequent 5 years using the presence/absence of an incident in each planning unit between 2009 and 2014 as a response variable. The model fitted a logistic curve using (bound by the years 1999–2008) the following: the number of previous incidents (probability of future conflict, logistic regression, *z* = 37.44, *n* = 335 694, *P* < 0.001), the number of fatalities (likelihood of conflict, *z* = 25.51, *P* < 0.001) and years since the last incident (probability of future conflict, logistic regression, *z* = 25.54, *n* = 335 694, *P* < 0.001). The logistic model generated probabilistic outputs (conflict-risk estimates between 1 and 100%). The model accounted for 35% of the total variation in the data (calculated using McFadden’s pseudo-R2). Following parameterization of the logistic model, conflict data from the years 2005 to 2014 predict the risk of conflict incidents during the next 5 years (2015–2019). For more details, see (Hammill et al. 2016). We resampled the model from 3 km^2^ to a resolution of 1 km^2^ to make it compatible with the scale of our analysis.

To map potential ASM threats to biodiversity, we used a freely available mining location point information dataset that was recently updated for the eastern DRC (International Peace Information Service (IPIS) 2019). This dataset includes information on the location of over 2400 mining sites, with data collected by IPIS field teams that visit each mining site and record data on mobile devices with satellite communicators (Matthysen et al. 2019). Many such mines are remote—at least 39% can only be accessed by local villages after at least two hours of walking (Matthysen et al. 2019). Mines represent not only an employment resource for locals, but also a resource for locals to trade and sell products to diggers, including bushmeat. It is increasingly recognised that many threats such as mining and infrastructure development impact the environment not only in their immediate vicinity, but also they can diffuse throughout the landscape (Tulloch et al. 2019). To represent the potential diffusion of impacts on biodiversity and ecosystem services from each mining point, we therefore placed a 20 km buffer around each mine location and developed a raster layer with impacts diminishing linearly (from 100% degraded at the point of each mine to 0% degraded at 20 km distant from the point). A 20 km threshold of impact was selected during a consultation workshop carried out in September 2017 with DRC conservation managers, government officials, and representatives from non-governmental organisations (Tulloch and Grantham 2019). This distance represents the furthest that a person living in or near a mining settlement is likely to travel to harvest resources (e.g. firewood, bushmeat) on a daily basis. Given this is the extent of our knowledge pertaining to diffuse ASM hunting impacts within the region, we are limited to assuming a commonly used linear relationship between the activity and its relative impact on biodiversity (Juffe-Bignoli et al. 2021). A full list of data included in the analysis is presented in Table 1.

**Table 1:** Maps of conservation features and threatening processes included in the spatial prioritisation with Zonation

Feature type	Number included in prioritisation	Details	Resolution and extent	Source
Species (modelled habitat suitability)	6	Presence or absence derived from species distribution models	1 km^2^, eastern DRC (Maiko-Itombwe Landscape)	Plumptre et al. (2016)
Species (modelled habitat suitability)	73	Presence or absence derived from species distribution models	300 m^2^, global	Rondinini et al. (2011)
Species (range)	8	Species presence or absence derived from species range maps	Vector, global	IUCN (2020b)
Species (occupancy model)	1 (Chimpanzee)	Probability of occurrence derived from species distribution model (0–0.97% probability)	30 m^2^, eastern DRC	Plumptre et al. (2015)
Ecosystems	20	Congo basin forest ecosystems presence or absence	Original sources used multiple resolutions ranging from 30–100 m^2^, Congo Basin	Shapiro et al. (2021)
Biomass	1	Above and belowground carbon density MgC ha^−1^	1 km^2^, global	Xu et al. (2017)
Current well-mapped threats	1	Forest Intactness data ranging between 0 and 100 which is a combination of datasets representing well-mapped anthropogenic threats to biodiversity including direct forest loss (Potapov et al. 2017), fragmentation (Shapiro et al. 2021), and human pressures such as access (Grantham et al. 2020a, b)	1 ha, eastern DRC	Grantham et al. (2020a, b)
Future well-mapped threats	1	A modelled value between 0 and 1 that represents the likelihood of an area being subject to deforestation	30 m^2^, eastern DRC (Maiko-Itombwe Landscape)	Goldman et al. (2017)
Mining	1	A modelled value representing diminishing impacts on biodiversity from human exploitation of the environment for ASM, up to 20 km from the point of each mine	Point data, eastern DRC	International Peace Information Service (IPIS) (2019) and Matthysen et al. (2019)
Human warfare conflict	1	A modelled value representing the likelihood of conflict occurring	3 km^2^, Africa	Hammill et al. (2016)

### Spatial prioritisation

We used the conservation planning software Zonation to identify the conservation priorities (Moilanen et al. 2014). Zonation iteratively ranks a series of units, in our case 1 km^2^ planning units, from lowest to highest priority for conservation. We applied the core-area Zonation algorithm, which iteratively removes cells that minimise biological loss. This is achieved by selecting and “removing” from the remaining priority cells at each iteration with the smallest occurrence for the most valuable feature over all conservation values in the cell (Moilanen et al. 2014). In the assessment of conservation priorities, all features were weighted equally (i.e. allocated a conservation weighting of 1), and no cost layer was used. We accounted for the impacts of future threats to biodiversity and carbon through Zonation’s condition and retention layers described below. From each scenario, we defined priority areas as the highest ranked 30% of the landscape and assessed this set of priorities against other scenarios.

### Scenarios

We explored seven scenarios that identify priority locations for conservation action using different broad strategies around ignoring, avoiding, and accounting for threats (Fig. 2; Table 2). We first explored a baseline threat-ignorant scenario (S1), which ignores ASM and armed conflict threats, accounting only for well-mapped threats. This baseline represents a commonly used scenario in conservation prioritisation (Menon et al. 2001; Laumonier et al. 2010; Fagundes et al. 2018). The well-mapped threat data (described above) was used as a condition layer in Zonation (Moilanen et al. 2014), representing the fraction of suitable habitat that remains for the biodiversity features in each grid cell after considering current and future well-mapped threats, across all scenarios across all features. We then explored a threat-avoiding management strategy that *avoids* the threats of artisanal mining (S2a), conflict (S3a) and both simultaneously (S4a), and a threat-accounting management strategy that *accounts* for the threats of artisanal mining (S2b), conflict (S3b) and both simultaneously (S4b). It is important to note that priorities were allocated based on the assumption that it is possible for a conservation action to achieve a positive outcome for biodiversity, of which the benefit may be immediate or long term. For example, when avoiding threats, the action may be to designate a protected area (increases in species populations) in a place where no or few threats exist. In comparison, accounting for a threat would assign management action such as restoration (once the threat has moved on) directly to locations that have been impacted and degraded, but would have high benefits for biodiversity if restored. Alleviating ASM and armed conflict threats to biodiversity in the DRC is not feasible, therefore targeting actions towards these threats may include restoration of priority areas once the threatening activity has finished or moved to a new location, or preventing bush meat hunting and incentivising companies to make food available to workers. Because the focus of our analysis is on accounting for and assessing management strategies to address the uncertain threats of armed conflict and ASM, we do not explore different management strategies for well-mapped threats such as direct habitat loss and proximity to human settlements.

**Table 2:** Scenario descriptions, respective management strategy to future mining and conflict threats and relevant data inclusions within the Zonation analysis undertaken across the eastern Democratic Republic of the Congo

Scenario	Management strategy to future mining and conflict threats	Condition layer used	Retention layer used
S1	Baseline—Ignorant to mining and conflict threats	Baseline condition (well-mapped threats)	NA
S2a	Avoid mining threats (ignore conflict)	Baseline condition (well-mapped threats) and mining threats	NA
S2b	Account for mining threats (ignore conflict)	Baseline condition (well-mapped threats) and mining threats	Mining
S3a	Avoid conflict threats (ignore mining)	Baseline condition (well-mapped threats) and conflict threats	NA
S3b	Account for conflict threats (ignore mining)	Baseline condition (well-mapped threats) and conflict threats	Conflict
S4a	Avoid mining and conflict threats	Baseline condition (well-mapped threats), conflict and mining threats	NA
S4b	Account for mining and conflict threats	Baseline condition (well-mapped threats), conflict and mining threats	Conflict and mining

### Threat-avoiding and threat-accounting management strategies

When avoiding ASM and conflict threats (scenarios 2a, 3a and 4a), we assume biodiversity persistence is lower where there is a threat present and avoid degraded areas when allocating conservation resources. To achieve this, we incorporated the respective threat into the prioritisation as a condition layer, thereby multiplying the value for each feature in each cell by the condition value. For each management strategy and scenario, condition layers are created by multiplying normalised (0–1) threat rasters together. Condition normalises present landscape condition values (threat layers) to be measured against a historical baseline (if there were no threats). Because Zonation removes cells with lower values first from the prioritisation, cells with higher priority will tend to avoid mining and conflict threats to conservation values, as prioritised cells will tend to have lower threat and higher condition (unless high threat intensity coincides with high biodiversity value).

When accounting for threats (scenarios 2b, 3b and 4b), we assumed that conservation actions could improve outcomes for biodiversity where a given threat is present. We recognise that the complexity around conflict and mining threats means in reality a biodiversity benefit through a threat-accounting strategy may be unlikely, making a strategy that avoids threats more desirable. If targeting a threat were to fail, then the quantified benefit may in fact represent a biodiversity loss. Resources are prioritised towards actions that mitigate threats to biodiversity in “high-value” areas (e.g. restoration of degraded ecosystems (Strassburg et al. 2019)) but may also be allocated to protected area designation if a relatively unthreatened area is identified as having high biodiversity value. To achieve this, we incorporated the intensity and distribution of ASM and conflict threats as a retention layer and again combined through multiplication in the scenario that considers both. In Zonation, a retention layer describes the fraction of cell condition retained or increased (in the case of management gain mode). We use the management gain retention mode (mode 2) which means that condition goes up according to the retention values (Moilanen et al. 2014). For example, a value of 1 in a retention layer implies no change in condition even in the absence of condition, and 1.2 implies a 20% management gain through targeted action (Moilanen et al. 2014). To create retention layers, we took each respective threat layer and subtracted it from 1.9 (rather than 2, assuming that a degraded area can never be equal to its original condition (Crouzeilles et al. 2016)). Let’s say the threat of ASM was impacting an area at a value of 0.2 (on the 0 (degraded) to 1 (pristine) scale, this is relatively high). In this case, the area has lost 0.8 of its value, some of which can be regained through management. In order to consider this potential management gain in our prioritisation, we allocate this area a retention value of 1.7 (which is 1.9–0.2). In other words, we assume that this area can regain 70% of its original biodiversity value through management. Any value less than 1 was allocated a value of 1 (as there is little to no threat here, there is no improvement to be made with action). An assumption of our analysis is that threats impact the biodiversity features equally, and that the impact (in the case of condition) and management benefit (in the case of retention) is relative to the afore-described values. For further technical explanations on condition and retention layers, please refer to the Zonation User Manual (Moilanen et al. 2014).

## Results

### Priority areas for conservation action

**Fig. 3:**
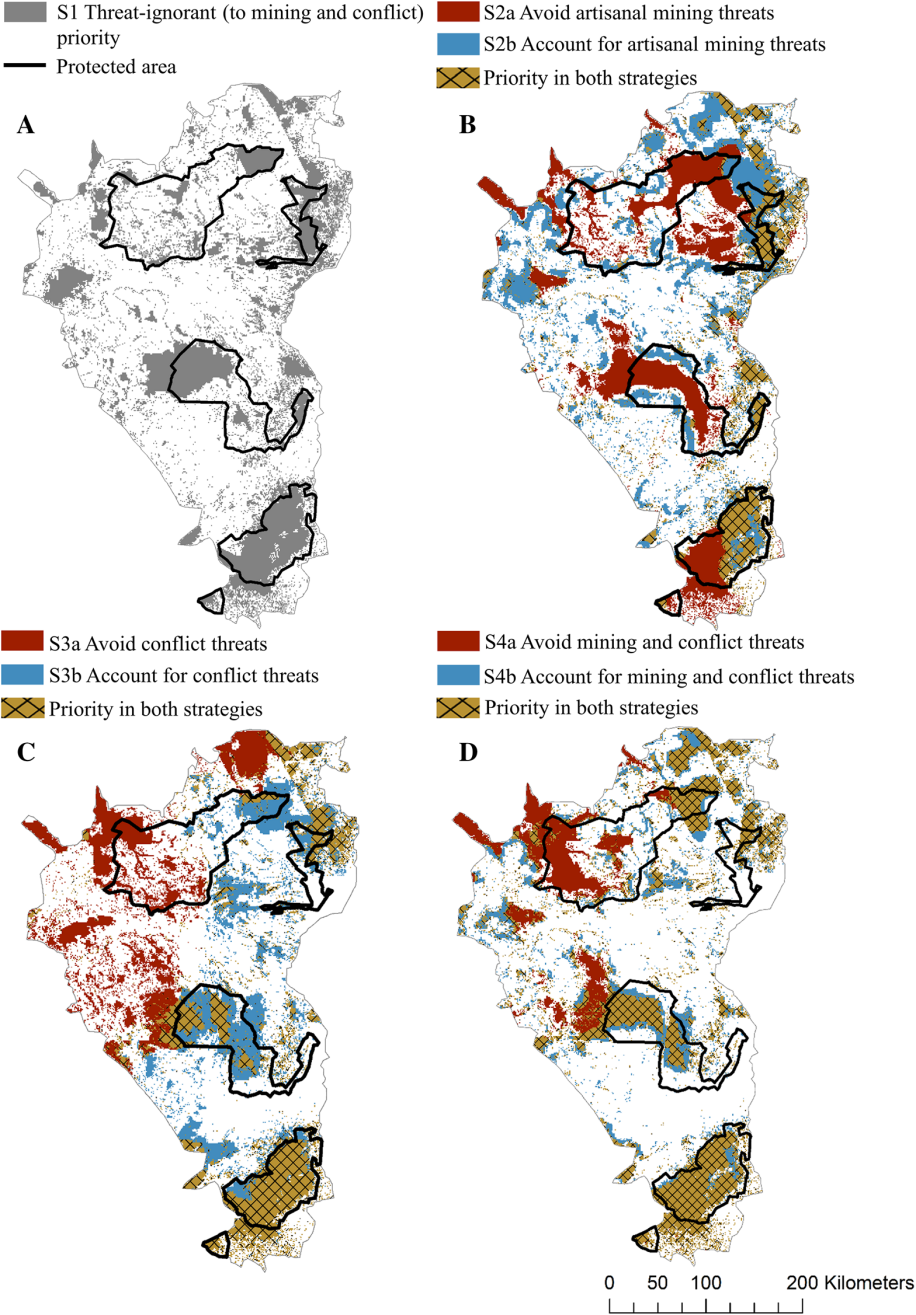
Priority areas (highest ranked 30% of the landscape) for **A** the threat-ignorant baseline scenario that ignores mining and conflict threats (S1), **B** the scenario that considers threats to biodiversity from diffuse mining impacts using a threat-avoiding (S2a) or threat-accounting (S2b) management strategy, **C** the scenario that considers threats to biodiversity from conflict impacts using a threat-avoiding (S3a) or threat-accounting (S3b) management strategy and **D** the scenario that considers threats to biodiversity from both diffuse mining and conflict impacts using a threat-avoiding (S4a) or threat-accounting (S4b) management strategy. Priority areas using threat-avoiding strategies are shown in red, threat-accounting strategies are shown in blue, and priority areas selected both strategies are shown in brown (hatched). Current protected areas are outlined in black

We found that ignoring, targeting or accounting for mining and conflict threats yields vastly different spatial priority solutions (Fig. 3). Ignoring mining and conflict threats was especially problematic as it missed between 16 300 km^2^ and 23 600 km^2^ of areas that were identified as priorities when they were considered (across all strategies and scenarios), which may compromise any conservation action taken that does not specifically account for the threats. We also found that considering threats to conservation features independently rather than simultaneously potentially misses benefits to 13 800–14 800 km^2^, and 15 700–25 100 km^2^ in the threat-avoiding and threat-accounting scenarios, respectively (Fig. 3). The far south-eastern and north-eastern parts of the study region, areas within the Albertine Rift, were selected as priorities in all scenarios under all management strategies (Fig. 3).

The existing level of protection of priority cells for strategies accounting for threats in spatial conservation prioritisation varied across scenarios. The strategy that accounts for both mining and conflict threats (S4b—46.6%) had the most amount of priority areas inside of current protected areas, followed by the strategy that avoids them (S4a—42.3%). This was closely followed by the strategy that avoids only mining threats (S2a—42.1%), the strategy that is ignorant to both mining and conflict threats (Baseline—41.6%), the strategy that accounts for only conflict threats (S3b—41.5%) and by the strategy that avoids only conflict threats (S3b—40.4%). Interestingly, the strategy of only accounting for mining threats indicated that the current protected area network is not well suited to this goal, with the least amount of priority areas in current protected areas (S2b—28.1%).

### Differences among solutions

**Fig. 4:**
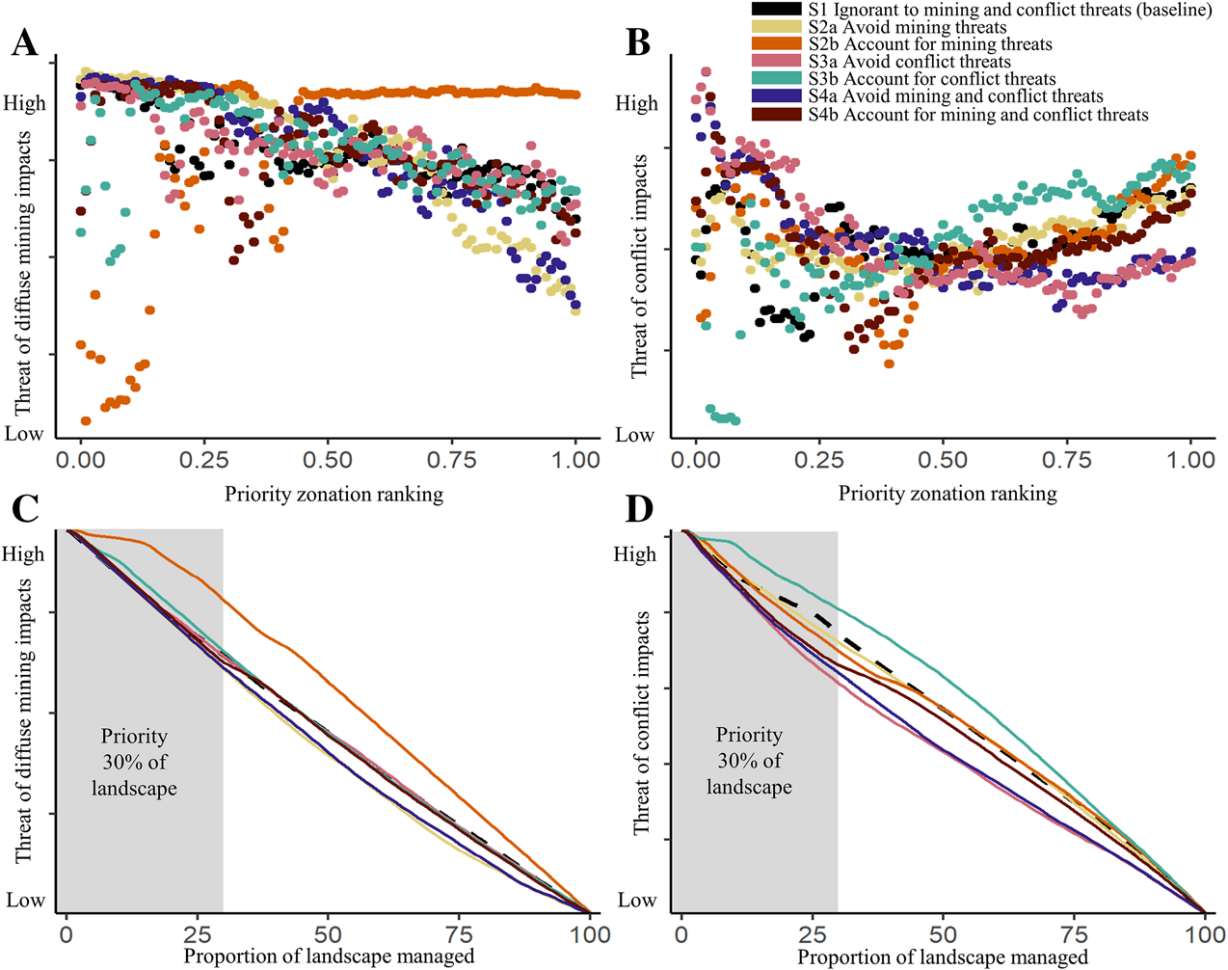
Panels **A** and **B** show the average risk to features from diffuse mining and conflict impacts, respectively, at each 0.01 increment of the zonation ranking. Panels **C** and **D** show the total risk from diffuse mining and conflict impacts, respectively, at each proportion of landscape managed, with the baseline shown in a dashed line. Here, management refers to any conservation action (such as protection or enhanced regulation). If the objective of a decision maker was to reduce the impact of a threat, the threat-*accounting* management strategy represents how much a threats impact could be reduced by assuming unlimited resources. However, if a threat rendered an area too dangerous, or severely degraded with little conservation value, the threat-*avoiding* strategy represents how much of a threat could be avoided at each ranking (**A** and **B**), or cumulatively for the proportion of landscape managed (**C** and **D**). Priority locations are highlighted in grey of **C** and **D**

Intuitively, a threat-*avoiding* management strategy results in less risk of diffuse mining and conflict impacts on conservation features, when compared to a threat-*accounting* management strategy (grey shaded areas in Fig. 4).

**Fig. 5:**
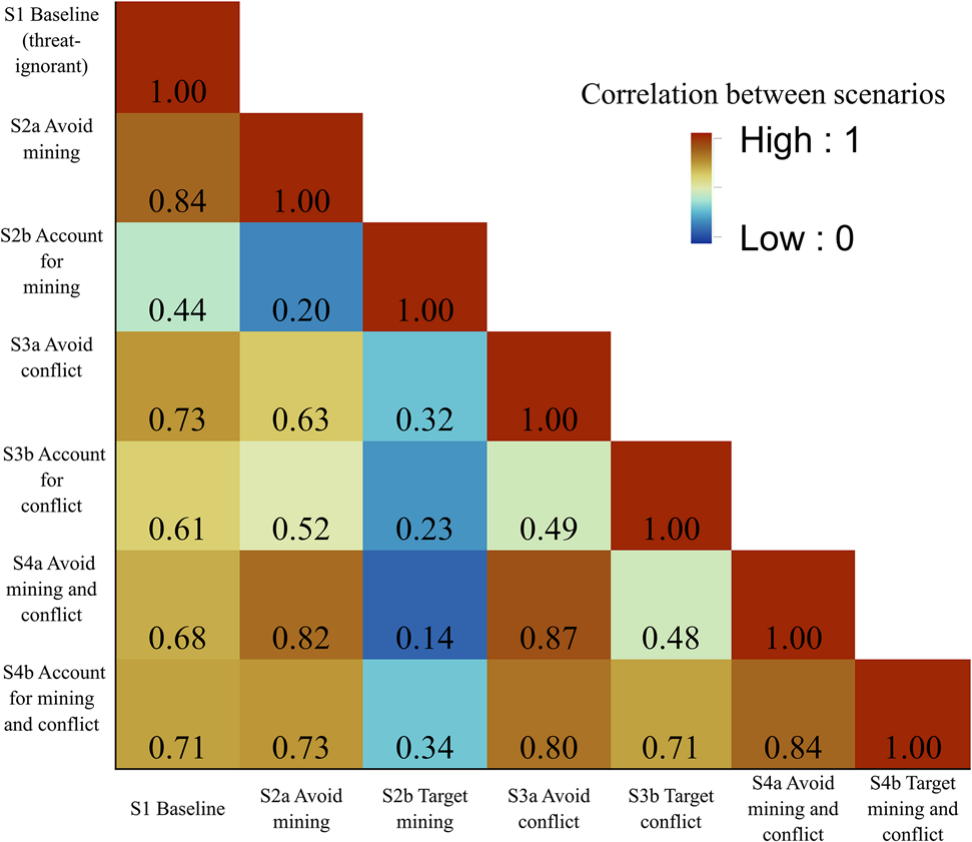
Correlation matrix showing the Pearson correlation coefficient between each ranked output. Values were calculated using the Band Collection Statistics tool in ArcMap version 10.5 (ESRI 2017)

The ranked outputs for the strategy of accounting for only mining threats were least correlated with the baseline (S2b; Fig. 5), which indicates that ignoring mining when designating where to allocate conservation resources might miss important locations for biodiversity recovery and persistence. The spatial prioritisation outputs that differed the most from one another were avoiding threats from both mining and conflict, and accounting for only mining (Pearson correlation coefficient 0.14, S4a and S2b, Fig. 5). All outputs associated with the strategy of accounting for priorities towards artisanal mining were poorly correlated with any other output, indicating that in general, considering threats from artisanal mining has a bigger impact on changing spatial priorities than threats from conflict (Fig. 5). In contrast, the ranked outputs for the strategy of avoiding threats of mining only, conflict only, or mining and conflict were more correlated with the baseline (threat-ignorant) strategy, which ignores these threats (Pearson correlation coefficients 0.84, 0.73 and 0.68, respectively; Fig. 5).

### Representation of features

**Fig. 6:**
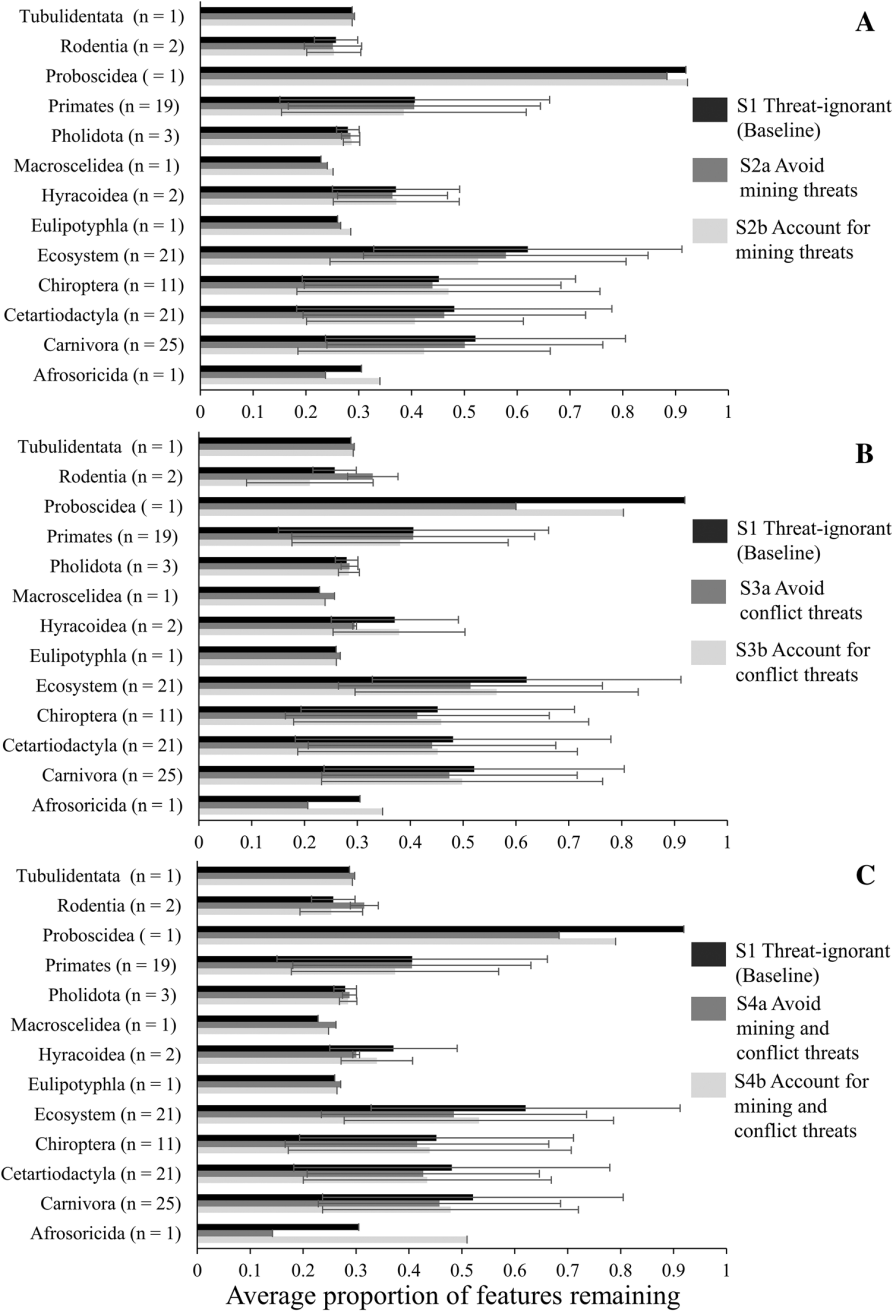
Average proportion of range remaining for each species order/feature type under each scenario in priority conservation areas (top 30% of zonation ranking), regardless of protection status. That is for scenarios that consider associated impacts of **A** mining, **B** conflict and **C** both mining and conflict simultaneously, using threat-ignorant, threat-avoiding and threat-accounting management strategies. Error bars represent standard deviation

When mining and conflict threats are ignored, in most cases, there is a higher representation of features in priority conservation areas (Baseline or threat-ignorant scenario; Fig. 6), indicating that a management strategy that ignores threats comes with a high risk of impacts to features from mining and conflict threats. When the single mining threat is considered, a higher proportion of features’ ranges on average remains in the priority conservation areas (top 30% of Zonation ranking) using the threat-*accounting* strategy (62% of orders/feature types) compared to a threat-*avoiding* strategy, exceptions being *Carnivora, Cetartiodactyla, Ecosystems*, and *Primates* (Fig. 6). When the single conflict threat is considered, a higher proportion of features ranges on average remain in the priority conservation areas (top 30% of Zonation ranking) again using the threat-*accounting* strategy (53%), but this time, the exceptions are *Eulipotyphla, Macroscelidea, Pholidota, Primates* (again)*, Rodentia* and *Tubulidentata.* When both mining and conflict threats are considered simultaneously, these are the same orders/feature types that had a higher proportion remaining in priority areas in the threat-*accounting* strategy. This indicates that in general accounting for the threats directly in addition to protective mechanisms will have greater biodiversity benefits than avoiding the threats, however, not for every species/feature type.

Under the threat-*avoiding* strategy, where the risk of mining and conflict threat was considered, representation of the order *Afrosoricida* (shrews) in priority conservation locations declined most (53.5%) and representation of the order *Rodentia* (rodents) increased the most (28.1%) when compared to the baseline. Under the threat-*accounting* strategy, representation of the order *Carnivora* (carnivores) in priority conservation locations declined the most under the mining-associated threat scenario (by 18.7%), and the order *Afrosoricida* (shrews) increased the most (53.5%) in the scenario where both threats are considered simultaneously (Fig. 6).

## Discussion

In the eastern DRC, areas that host important biodiversity, and are threatened by land-use change and associated habitat loss, are also exposed to diffuse impacts associated with localised artisanal mining and conflict activities. We found that by applying a management strategy that is ignorant to mining and conflict threats, a substantial area of our study region (16 300–23 600 km^2^; or 16.5–23.8% of the study region) was potentially mis-identified as a priority for conservation action. We have focussed on the implications of different management strategies for accounting for mining and conflict threats; however, our results highlight the importance of accounting for all known threats in conservation prioritisation, including those not accounted for here (Benítez-López et al. 2019), as a threat-ignorant management strategy risks unsuccessful conservation outcomes in these areas.

Beyond evaluating the impact of particular threats on spatial conservation priorities, our analysis shows that it is crucial to account for all threats simultaneously within a landscape when conducting land-use planning and conservation prioritisation. We found that when considering both mining and conflict threats, using a threat*-accounting* management strategy mostly resulted in a higher representation of features in high-priority locations than a threat-*avoiding* strategy (Fig. 6). When at least one threat is ignored, conservation priorities for single-threat strategies are placed in direct conflict with the ignored threat (Fig. 4). Often mining and conflict threats overlap and in some cases influence each other (Butsic et al. 2015); however, in some cases, avoiding the threat of conflict but ignoring the threat of mining results in high-priority locations with high mining impacts, while avoiding mining but ignoring the threat of conflict resulted in high-priority locations with high conflict probability (Fig. 4). We therefore recommend that, where feasible, and safe and effective to do so, a threat-accounting management strategy within identified priority areas, which considers both well-mapped and uncertain diffuse mining and conflict threats, may be the most effective strategy for biodiversity representation in priority areas for conservation. However, we note that for species-specific conservation objectives, the most effective strategy will vary (Fig. 6).

Our results align with previous studies which have shown that risk-aversion and avoidance of threatening processes can result in suboptimal conservation prioritisation solutions. Hammill et al. (2016) found that across Africa, avoiding the risk posed to biodiversity from conflict when designating protected areas results in lower protection of species and the lowest return on investment when compared to conflict-ignorant, conflict-accounting and conflict-sensitive management strategies. In New Zealand, avoiding the risk of failed management meant that fewer threatened species could be recovered compared to a risk-accounting strategy (Tulloch et al. 2015a, b). Despite the potential for poorer biodiversity outcomes, conservation practitioners are often risk averse due to repercussions of failed projects (e.g. in terms of continued funding from donors) and often opt for sub-optimal lower risk actions in place of higher-risk actions with higher potential biodiversity benefits (Maguire and Albright 2005). Risk analysis, or decision analysis, that quantifies organisational risk tolerance level so that it can be incorporated into decision support frameworks, is increasingly promoted as a way to help overcome this (Maguire 1991; Wilson et al. 2005a, b; Tulloch et al. 2015a, b).

That being said, some conservation actions are riskier than others. In the study region, lives can be lost carrying out conservation actions, meaning that some areas must be avoided at all costs (Plumptre 2003). Volatility in the region means that there are many socio-political factors at play that must be considered when implementing conservation actions, some of which are not considered in our spatial prioritisation and are far beyond the scope of this paper (Draulans and Van Krunkelsven 2002; Plumptre 2003). Our approach, and other advances on it, could be used to identify priorities that result in improved biodiversity outcomes, by reflecting what actions are safe to carry out; for example, it could be used to “target” mining threats (through interventions which may reduce impacts in the long run) while “avoiding” armed conflict threats altogether. Clearly defining how threats are going to be treated (avoided, accounted for and/or ignored) is key, as the results of spatial prioritisations can vary widely depending on how they are treated as demonstrated here.

Effective management of threats requires substantial investment in actions and policies beyond designation of protected areas and implementation of particular management actions within these areas such as anti-poaching patrols (Walpole and Wilder 2008). At least some level of threat of conflict due to human warfare and mining impacts occurs across most of the study region, 99.99% and 83.81%, respectively. Faced with the broad and unavoidable nature of these threats, actions to recover biodiversity in the region must include other area-based conservation measures (Dudley et al. 2018), such as restoration of a site once a threat has been alleviated (if it still has a high capacity to support biodiversity) (Festin et al. 2019). Targeted conservation actions to reduce the impacts of ASM include payment schemes for ecosystem services (Bofin et al. 2011), or socio-economic reforms such as the development of alternative livelihoods for local people who are faced with few choices than to join armed groups or work in artisanal mines (Geenen and Radley 2014; IGF 2017). While conflict mitigation in conservation is often unachievable, some targeting strategies where it is safe and feasible include impact assessment and response of vulnerable areas, maintaining management capacity, maintaining a presence, supporting staff, maintaining neutrality and sourcing external funding and finance support replacing those that are no longer in place (Oglethorpe et al. 2004).

In all scenarios and under all management strategies, the eastern side of the study region, where the Albertine Rift is situated, was identified as a high priority (Fig. 3). The Albertine Rift is an incredibly important region for biodiversity and carbon sequestration (Plumptre et al. 2007). These areas are critical for conservation action due to their high endemism and biodiversity richness. They are robust to management strategy choice and are high priorities despite multiple threats that coincide there—in particular, encroachment of human development and resource use. Many parts of this region have already been cleared and are at risk from further deforestation due to their fertile soils that are valuable for agriculture (Ryan et al. 2017). The challenge for conservation practitioners and decision makers will be to discover and implement effective conservation mechanisms that enable the thousands of people living in the Albertine Rift to live healthily, sustainably and safely in the landscapes to ensure biodiversity and human livelihoods are maintained.

Given the importance of mining and conflict threats in driving spatial conservation priorities in the region, there is an urgent need for better understanding of the threats to biodiversity that enable existing models of impact spread, magnitude and longevity to be improved. We have used proxy data to represent these threats, each containing inherent bias (for example, some mining locations remain unknown), but models will be improved when ground-truthed impact assessments from similar locations are conducted and incorporated (e.g. Rahm et al. 2015). Additionally, we assumed that the threats included in our analysis impact biodiversity features equally; however, in reality, the impact of different threats on a species is highly variable, and different species are also likely to respond to how threats are managed in different ways (Carwardine et al. 2012). This is an important area of active research (Tulloch et al. 2018) and our results could be improved when regional information on species-specific responses to threats become available in the future.

Our results could be improved in the future by refined data on the distributions of species and threats to species. We included the best species distribution data available, and those listed as threatened by hunting by the IUCN; however, in some cases, these distributions and threats may be over- or under-represented across the landscape. In this analysis, we considered only biophysical conservation features (hunted mammals, ecosystems and carbon). Beyond the scope of this study, there are additional socio-economic objectives, drivers of change and trade-offs with biodiversity conservation, which must be considered before developing a comprehensive conservation plan (Burnley 2012). Specifically, indigenous and marginalised communities must be involved when developing a conservation plan, to ensure that conservation actions account for local interests, such as traditional livelihoods (Cuni-Sanchez et al. 2019). Examples of conservation approaches in the region that attempt to address multiple objectives for biodiversity and traditional livelihoods include community-driven conservation planning (Nackoney et al. 2013), alternative livelihoods strategies (Cuni-Sanchez et al. 2019), and sustainable agroforestry (Dumont et al. 2019).

We did not include the cost of managing biodiversity under our threat-avoiding versus threat-accounting strategies as the range of mechanisms for each strategy is wide and the costs are hugely variable and poorly known. For example, although “avoiding” a threat could be enacted simply by designating a protected area in a location with high biodiversity value away from that threat, an alternative action might be to allocate more resources to the management of people and threats (e.g. invasive species, disease) in existing protected areas. An important next step would be to identify specific actions necessary to enhance biodiversity in the region for each spatial solution. Additionally, we assumed that the impact of a threat on conservation features is relative to the intensity of the threat (Tallis et al. 2008). Conservation priorities would be greatly improved if there were better information on the impact of the severity of threats on biodiversity, which could be facilitated through improved species monitoring (Nicol et al. 2019). Another potential limitation was the 20 km buffer used to designate localised ASM impacts, where we assumed based on expert input that there is no risk to biodiversity conservation outcomes beyond this point. Future studies could evaluate how further diffusion of impacts beyond the 20 km buffer might affect conservation outcomes, or how different models of threat diffusion might change the spatial location of priority areas for biodiversity conservation (Tulloch et al. 2019). We used the best and most up-to-date datasets available for the region, but these use different mapping techniques and are at different resolutions. In the future, improved and more congruent datasets could improve our results. Finally, the representation of ASM and armed conflict in this analysis is a simplistic estimation of the potential impacts on biodiversity. As the locations of conflict incidents and mines are the only impacts that are mapped, we use the afore-described spatial layers (“Threats” section) to determine if and by how much their inclusion may change the outcomes of a spatial prioritisation. Further research and data are required to further map or model these impacts.

## Conclusion

We have shown that compared to only accounting for well-mapped threats, accounting for uncertain threats of ASM and armed conflict drastically changes conservation priorities. For maintaining and recovering biodiversity in the eastern DRC, specifically accounting for and targeting all threats will likely result in better conservation outcomes (assuming that the impact to biodiversity from these threats can be reduced) than one that avoids acting in areas where at least one known threat occurs. Evaluating a variety of spatial prioritisation solutions that show the implications of considering threats in different ways adds transparency to the decision-making process, which is particularly important in landscapes such as the DRC where the risks of mis-allocated resources to both biodiversity and people are significant, and the biodiversity stakes are high.

## Supplementary Information

Below is the link to the electronic supplementary material.

Supplementary file1 (PDF 934 kb)
