# Supplementary material for: Reconsidering priorities for forest conservation when considering the threats of mining and armed conflict
Source: Ambio. 2022 Apr 10;51(9):2007–24. doi: 10.1007/s13280-022-01724-0 (PMC9287519; doi:10.1007/s13280-022-01724-0)
Supplement: Supplementary file 1 — Supplementary file1 (PDF 934 kb) [file 13280_2022_1724_MOESM1_ESM.pdf]

Ambio

Supplementary Information

This supplementary information has not been peer reviewed.

Title: **Reconsidering priorities for forest conservation when considering the threats of mining and armed conflict**

Appendix A – Complete list of included features

Features	Order/Type	Source of spatial data
Potamogale velox	Afrosoricida	Rondinini, C., Di Marco, M., Chiozza, F., Santulli, G., Baisero, D., Visconti, P., Hoffmann, M., Schipper, J., Stuart, S.N., Tognelli, M.F., 2011. Global habitat suitability models of terrestrial mammals. Philos. Trans. R. Soc. B Biol. Sci. 366, 2633–2641.
Crocuta crocuta	Carnivora	Plumptre, A.J., Ayebare, S., Segan, D., Watson, J. & Kujirakwinja, D., 2016. Conservation Action Plan for the Albertine Rift. Rep. Wildl. Conserv. Soc. its Partners.
Panthera leo	Carnivora	Plumptre, A.J., Ayebare, S., Segan, D., Watson, J. & Kujirakwinja, D., 2016. Conservation Action Plan for the Albertine Rift. Rep. Wildl. Conserv. Soc. its Partners.
Aonyx congicus	Carnivora	Rondinini, C., Di Marco, M., Chiozza, F., Santulli, G., Baisero, D., Visconti, P., Hoffmann, M., Schipper, J., Stuart, S.N., Tognelli, M.F., 2011. Global habitat suitability models of terrestrial mammals. Philos. Trans. R. Soc. B Biol. Sci. 366, 2633–2641.
Canis adustus	Carnivora	Rondinini, C., Di Marco, M., Chiozza, F., Santulli, G., Baisero, D., Visconti, P., Hoffmann, M., Schipper, J., Stuart, S.N., Tognelli, M.F., 2011. Global habitat suitability models of terrestrial mammals. Philos. Trans. R. Soc. B Biol. Sci. 366, 2633–2641.
Caracal caracal	Carnivora	Rondinini, C., Di Marco, M., Chiozza, F., Santulli, G., Baisero, D., Visconti, P., Hoffmann, M., Schipper, J., Stuart, S.N., Tognelli, M.F., 2011. Global habitat suitability models of terrestrial mammals. Philos. Trans. R. Soc. B Biol. Sci. 366, 2633–2641.
Leptailurus serval	Carnivora	Rondinini, C., Di Marco, M., Chiozza, F., Santulli, G., Baisero, D., Visconti, P., Hoffmann, M., Schipper, J., Stuart, S.N., Tognelli, M.F., 2011. Global habitat suitability models of terrestrial mammals. Philos. Trans. R. Soc. B Biol. Sci. 366, 2633–2641.
Hydrictis maculicollis	Carnivora	Rondinini, C., Di Marco, M., Chiozza, F., Santulli, G., Baisero, D., Visconti, P., Hoffmann, M., Schipper, J., Stuart, S.N., Tognelli, M.F., 2011. Global habitat suitability models of terrestrial mammals. Philos. Trans. R. Soc. B Biol. Sci. 366, 2633–2641.
Genetta piscivora	Carnivora	Rondinini, C., Di Marco, M., Chiozza, F., Santulli, G., Baisero, D., Visconti, P., Hoffmann, M., Schipper, J., Stuart, S.N., Tognelli, M.F., 2011. Global habitat suitability models of terrestrial mammals. Philos. Trans. R. Soc. B Biol. Sci. 366, 2633–2641.

Panthera pardus	Carnivora	Rondinini, C., Di Marco, M., Chiozza, F., Santulli, G., Baisero, D., Visconti, P., Hoffmann, M., Schipper, J., Stuart, S.N., Tognelli, M.F., 2011. Global habitat suitability models of terrestrial mammals. Philos. Trans. R. Soc. B Biol. Sci. 366, 2633–2641.
Caracal aurata	Carnivora	Rondinini, C., Di Marco, M., Chiozza, F., Santulli, G., Baisero, D., Visconti, P., Hoffmann, M., Schipper, J., Stuart, S.N., Tognelli, M.F., 2011. Global habitat suitability models of terrestrial mammals. Philos. Trans. R. Soc. B Biol. Sci. 366, 2633–2641.
Atilax paludinosus	Carnivora	Rondinini, C., Di Marco, M., Chiozza, F., Santulli, G., Baisero, D., Visconti, P., Hoffmann, M., Schipper, J., Stuart, S.N., Tognelli, M.F., 2011. Global habitat suitability models of terrestrial mammals. Philos. Trans. R. Soc. B Biol. Sci. 366, 2633–2641.
Bdeogale nigripes	Carnivora	Rondinini, C., Di Marco, M., Chiozza, F., Santulli, G., Baisero, D., Visconti, P., Hoffmann, M., Schipper, J., Stuart, S.N., Tognelli, M.F., 2011. Global habitat suitability models of terrestrial mammals. Philos. Trans. R. Soc. B Biol. Sci. 366, 2633–2641.
Crossarchus alexandri	Carnivora	Rondinini, C., Di Marco, M., Chiozza, F., Santulli, G., Baisero, D., Visconti, P., Hoffmann, M., Schipper, J., Stuart, S.N., Tognelli, M.F., 2011. Global habitat suitability models of terrestrial mammals. Philos. Trans. R. Soc. B Biol. Sci. 366, 2633–2641.
Herpestes sanguineus	Carnivora	Rondinini, C., Di Marco, M., Chiozza, F., Santulli, G., Baisero, D., Visconti, P., Hoffmann, M., Schipper, J., Stuart, S.N., Tognelli, M.F., 2011. Global habitat suitability models of terrestrial mammals. Philos. Trans. R. Soc. B Biol. Sci. 366, 2633–2641.
Herpestes ichneumon	Carnivora	Rondinini, C., Di Marco, M., Chiozza, F., Santulli, G., Baisero, D., Visconti, P., Hoffmann, M., Schipper, J., Stuart, S.N., Tognelli, M.F., 2011. Global habitat suitability models of terrestrial mammals. Philos. Trans. R. Soc. B Biol. Sci. 366, 2633–2641.
Herpestes naso	Carnivora	Rondinini, C., Di Marco, M., Chiozza, F., Santulli, G., Baisero, D., Visconti, P., Hoffmann, M., Schipper, J., Stuart, S.N., Tognelli, M.F., 2011. Global habitat suitability models of terrestrial mammals. Philos. Trans. R. Soc. B Biol. Sci. 366, 2633–2641.
Ichneumia albicauda	Carnivora	Rondinini, C., Di Marco, M., Chiozza, F., Santulli, G., Baisero, D., Visconti, P., Hoffmann, M., Schipper, J., Stuart, S.N., Tognelli, M.F., 2011. Global habitat suitability models of terrestrial mammals. Philos. Trans. R. Soc. B Biol. Sci. 366, 2633–2641.

Mungos mungo	Carnivora	Rondinini, C., Di Marco, M., Chiozza, F., Santulli, G., Baisero, D., Visconti, P., Hoffmann, M., Schipper, J., Stuart, S.N., Tognelli, M.F., 2011. Global habitat suitability models of terrestrial mammals. Philos. Trans. R. Soc. B Biol. Sci. 366, 2633–2641.
Mellivora capensis	Carnivora	Rondinini, C., Di Marco, M., Chiozza, F., Santulli, G., Baisero, D., Visconti, P., Hoffmann, M., Schipper, J., Stuart, S.N., Tognelli, M.F., 2011. Global habitat suitability models of terrestrial mammals. Philos. Trans. R. Soc. B Biol. Sci. 366, 2633–2641.
Ictonyx striatus	Carnivora	Rondinini, C., Di Marco, M., Chiozza, F., Santulli, G., Baisero, D., Visconti, P., Hoffmann, M., Schipper, J., Stuart, S.N., Tognelli, M.F., 2011. Global habitat suitability models of terrestrial mammals. Philos. Trans. R. Soc. B Biol. Sci. 366, 2633–2641.
Poecilogale albinucha	Carnivora	Rondinini, C., Di Marco, M., Chiozza, F., Santulli, G., Baisero, D., Visconti, P., Hoffmann, M., Schipper, J., Stuart, S.N., Tognelli, M.F., 2011. Global habitat suitability models of terrestrial mammals. Philos. Trans. R. Soc. B Biol. Sci. 366, 2633–2641.
Civettictis civetta	Carnivora	Rondinini, C., Di Marco, M., Chiozza, F., Santulli, G., Baisero, D., Visconti, P., Hoffmann, M., Schipper, J., Stuart, S.N., Tognelli, M.F., 2011. Global habitat suitability models of terrestrial mammals. Philos. Trans. R. Soc. B Biol. Sci. 366, 2633–2641.
Genetta maculata	Carnivora	Rondinini, C., Di Marco, M., Chiozza, F., Santulli, G., Baisero, D., Visconti, P., Hoffmann, M., Schipper, J., Stuart, S.N., Tognelli, M.F., 2011. Global habitat suitability models of terrestrial mammals. Philos. Trans. R. Soc. B Biol. Sci. 366, 2633–2641.
Genetta victoriae	Carnivora	Rondinini, C., Di Marco, M., Chiozza, F., Santulli, G., Baisero, D., Visconti, P., Hoffmann, M., Schipper, J., Stuart, S.N., Tognelli, M.F., 2011. Global habitat suitability models of terrestrial mammals. Philos. Trans. R. Soc. B Biol. Sci. 366, 2633–2641.
Poiana richardsonii	Carnivora	Rondinini, C., Di Marco, M., Chiozza, F., Santulli, G., Baisero, D., Visconti, P., Hoffmann, M., Schipper, J., Stuart, S.N., Tognelli, M.F., 2011. Global habitat suitability models of terrestrial mammals. Philos. Trans. R. Soc. B Biol. Sci. 366, 2633–2641.
Hippopotamus amphibius	Cetartiodactyla	Plumptre, A.J., Ayebare, S., Segan, D., Watson, J. & Kujirakwinja, D., 2016. Conservation Action Plan for the Albertine Rift. Rep. Wildl. Conserv. Soc. its Partners.
Okapia johnstoni	Cetartiodactyla	Plumptre, A.J., Ayebare, S., Segan, D., Watson, J. & Kujirakwinja, D., 2016. Conservation Action Plan for the Albertine Rift. Rep. Wildl. Conserv. Soc. its Partners.

Cephalophus dorsalis	Cetartiodactyla	Rondinini, C., Di Marco, M., Chiozza, F., Santulli, G., Baisero, D., Visconti, P., Hoffmann, M., Schipper, J., Stuart, S.N., Tognelli, M.F., 2011. Global habitat suitability models of terrestrial mammals. Philos. Trans. R. Soc. B Biol. Sci. 366, 2633–2641.
Cephalophus leucogaster	Cetartiodactyla	Rondinini, C., Di Marco, M., Chiozza, F., Santulli, G., Baisero, D., Visconti, P., Hoffmann, M., Schipper, J., Stuart, S.N., Tognelli, M.F., 2011. Global habitat suitability models of terrestrial mammals. Philos. Trans. R. Soc. B Biol. Sci. 366, 2633–2641.
Philantomba monticola	Cetartiodactyla	Rondinini, C., Di Marco, M., Chiozza, F., Santulli, G., Baisero, D., Visconti, P., Hoffmann, M., Schipper, J., Stuart, S.N., Tognelli, M.F., 2011. Global habitat suitability models of terrestrial mammals. Philos. Trans. R. Soc. B Biol. Sci. 366, 2633–2641.
Cephalophus nigrifrons	Cetartiodactyla	Rondinini, C., Di Marco, M., Chiozza, F., Santulli, G., Baisero, D., Visconti, P., Hoffmann, M., Schipper, J., Stuart, S.N., Tognelli, M.F., 2011. Global habitat suitability models of terrestrial mammals. Philos. Trans. R. Soc. B Biol. Sci. 366, 2633–2641.
Cephalophus silvicultor	Cetartiodactyla	Rondinini, C., Di Marco, M., Chiozza, F., Santulli, G., Baisero, D., Visconti, P., Hoffmann, M., Schipper, J., Stuart, S.N., Tognelli, M.F., 2011. Global habitat suitability models of terrestrial mammals. Philos. Trans. R. Soc. B Biol. Sci. 366, 2633–2641.
Cephalophus weynsi	Cetartiodactyla	Rondinini, C., Di Marco, M., Chiozza, F., Santulli, G., Baisero, D., Visconti, P., Hoffmann, M., Schipper, J., Stuart, S.N., Tognelli, M.F., 2011. Global habitat suitability models of terrestrial mammals. Philos. Trans. R. Soc. B Biol. Sci. 366, 2633–2641.
Hyemoschus aquaticus	Cetartiodactyla	Rondinini, C., Di Marco, M., Chiozza, F., Santulli, G., Baisero, D., Visconti, P., Hoffmann, M., Schipper, J., Stuart, S.N., Tognelli, M.F., 2011. Global habitat suitability models of terrestrial mammals. Philos. Trans. R. Soc. B Biol. Sci. 366, 2633–2641.
Kobus ellipsiprymnus	Cetartiodactyla	Rondinini, C., Di Marco, M., Chiozza, F., Santulli, G., Baisero, D., Visconti, P., Hoffmann, M., Schipper, J., Stuart, S.N., Tognelli, M.F., 2011. Global habitat suitability models of terrestrial mammals. Philos. Trans. R. Soc. B Biol. Sci. 366, 2633–2641.
Kobus kob	Cetartiodactyla	Rondinini, C., Di Marco, M., Chiozza, F., Santulli, G., Baisero, D., Visconti, P., Hoffmann, M., Schipper, J., Stuart, S.N., Tognelli, M.F., 2011. Global habitat suitability models of terrestrial mammals. Philos. Trans. R. Soc. B Biol. Sci. 366, 2633–2641.

Neotragus batesi	Cetartiodactyla	Rondinini, C., Di Marco, M., Chiozza, F., Santulli, G., Baisero, D., Visconti, P., Hoffmann, M., Schipper, J., Stuart, S.N., Tognelli, M.F., 2011. Global habitat suitability models of terrestrial mammals. Philos. Trans. R. Soc. B Biol. Sci. 366, 2633–2641.
Redunca arundinum	Cetartiodactyla	Rondinini, C., Di Marco, M., Chiozza, F., Santulli, G., Baisero, D., Visconti, P., Hoffmann, M., Schipper, J., Stuart, S.N., Tognelli, M.F., 2011. Global habitat suitability models of terrestrial mammals. Philos. Trans. R. Soc. B Biol. Sci. 366, 2633–2641.
Sylvicapra grimmia	Cetartiodactyla	Rondinini, C., Di Marco, M., Chiozza, F., Santulli, G., Baisero, D., Visconti, P., Hoffmann, M., Schipper, J., Stuart, S.N., Tognelli, M.F., 2011. Global habitat suitability models of terrestrial mammals. Philos. Trans. R. Soc. B Biol. Sci. 366, 2633–2641.
Syncerus caffer	Cetartiodactyla	Rondinini, C., Di Marco, M., Chiozza, F., Santulli, G., Baisero, D., Visconti, P., Hoffmann, M., Schipper, J., Stuart, S.N., Tognelli, M.F., 2011. Global habitat suitability models of terrestrial mammals. Philos. Trans. R. Soc. B Biol. Sci. 366, 2633–2641.
Tragelaphus eurycerus	Cetartiodactyla	Rondinini, C., Di Marco, M., Chiozza, F., Santulli, G., Baisero, D., Visconti, P., Hoffmann, M., Schipper, J., Stuart, S.N., Tognelli, M.F., 2011. Global habitat suitability models of terrestrial mammals. Philos. Trans. R. Soc. B Biol. Sci. 366, 2633–2641.
Tragelaphus spekii	Cetartiodactyla	Rondinini, C., Di Marco, M., Chiozza, F., Santulli, G., Baisero, D., Visconti, P., Hoffmann, M., Schipper, J., Stuart, S.N., Tognelli, M.F., 2011. Global habitat suitability models of terrestrial mammals. Philos. Trans. R. Soc. B Biol. Sci. 366, 2633–2641.
Tragelaphus scriptus	Cetartiodactyla	Rondinini, C., Di Marco, M., Chiozza, F., Santulli, G., Baisero, D., Visconti, P., Hoffmann, M., Schipper, J., Stuart, S.N., Tognelli, M.F., 2011. Global habitat suitability models of terrestrial mammals. Philos. Trans. R. Soc. B Biol. Sci. 366, 2633–2641.
Hylochoerus meinertzhageni	Cetartiodactyla	Rondinini, C., Di Marco, M., Chiozza, F., Santulli, G., Baisero, D., Visconti, P., Hoffmann, M., Schipper, J., Stuart, S.N., Tognelli, M.F., 2011. Global habitat suitability models of terrestrial mammals. Philos. Trans. R. Soc. B Biol. Sci. 366, 2633–2641.
Potamochoerus larvatus	Cetartiodactyla	Rondinini, C., Di Marco, M., Chiozza, F., Santulli, G., Baisero, D., Visconti, P., Hoffmann, M., Schipper, J., Stuart, S.N., Tognelli, M.F., 2011. Global habitat suitability models of terrestrial mammals. Philos. Trans. R. Soc. B Biol. Sci. 366, 2633–2641.

Potamochoerus porcus	Cetartiodactyla	Rondinini, C., Di Marco, M., Chiozza, F., Santulli, G., Baisero, D., Visconti, P., Hoffmann, M., Schipper, J., Stuart, S.N., Tognelli, M.F., 2011. Global habitat suitability models of terrestrial mammals. Philos. Trans. R. Soc. B Biol. Sci. 366, 2633–2641.
Rhinolophus kahuzi	Chiroptera	IUCN, 2020. The IUCN Red List of Threatened Species. http://www.iucnredlist.org
Eidolon helvum	Chiroptera	Rondinini, C., Di Marco, M., Chiozza, F., Santulli, G., Baisero, D., Visconti, P., Hoffmann, M., Schipper, J., Stuart, S.N., Tognelli, M.F., 2011. Global habitat suitability models of terrestrial mammals. Philos. Trans. R. Soc. B Biol. Sci. 366, 2633–2641.
Epomops franqueti	Chiroptera	Rondinini, C., Di Marco, M., Chiozza, F., Santulli, G., Baisero, D., Visconti, P., Hoffmann, M., Schipper, J., Stuart, S.N., Tognelli, M.F., 2011. Global habitat suitability models of terrestrial mammals. Philos. Trans. R. Soc. B Biol. Sci. 366, 2633–2641.
Hipposideros ruber	Chiroptera	Rondinini, C., Di Marco, M., Chiozza, F., Santulli, G., Baisero, D., Visconti, P., Hoffmann, M., Schipper, J., Stuart, S.N., Tognelli, M.F., 2011. Global habitat suitability models of terrestrial mammals. Philos. Trans. R. Soc. B Biol. Sci. 366, 2633–2641.
Hypsignathus monstrosus	Chiroptera	Rondinini, C., Di Marco, M., Chiozza, F., Santulli, G., Baisero, D., Visconti, P., Hoffmann, M., Schipper, J., Stuart, S.N., Tognelli, M.F., 2011. Global habitat suitability models of terrestrial mammals. Philos. Trans. R. Soc. B Biol. Sci. 366, 2633–2641.
Micropteropus pusillus	Chiroptera	Rondinini, C., Di Marco, M., Chiozza, F., Santulli, G., Baisero, D., Visconti, P., Hoffmann, M., Schipper, J., Stuart, S.N., Tognelli, M.F., 2011. Global habitat suitability models of terrestrial mammals. Philos. Trans. R. Soc. B Biol. Sci. 366, 2633–2641.
Rhinolophus alcyone	Chiroptera	Rondinini, C., Di Marco, M., Chiozza, F., Santulli, G., Baisero, D., Visconti, P., Hoffmann, M., Schipper, J., Stuart, S.N., Tognelli, M.F., 2011. Global habitat suitability models of terrestrial mammals. Philos. Trans. R. Soc. B Biol. Sci. 366, 2633–2641.
Rousettus lanosus	Chiroptera	Rondinini, C., Di Marco, M., Chiozza, F., Santulli, G., Baisero, D., Visconti, P., Hoffmann, M., Schipper, J., Stuart, S.N., Tognelli, M.F., 2011. Global habitat suitability models of terrestrial mammals. Philos. Trans. R. Soc. B Biol. Sci. 366, 2633–2641.

Rousettus aegyptiacus	Chiroptera	Rondinini, C., Di Marco, M., Chiozza, F., Santulli, G., Baisero, D., Visconti, P., Hoffmann, M., Schipper, J., Stuart, S.N., Tognelli, M.F., 2011. Global habitat suitability models of terrestrial mammals. Philos. Trans. R. Soc. B Biol. Sci. 366, 2633–2641.
Macronycteris gigas	Chiroptera	Rondinini, C., Di Marco, M., Chiozza, F., Santulli, G., Baisero, D., Visconti, P., Hoffmann, M., Schipper, J., Stuart, S.N., Tognelli, M.F., 2011. Global habitat suitability models of terrestrial mammals. Philos. Trans. R. Soc. B Biol. Sci. 366, 2633–2641.
Rhinolophus ruwenzorii	Chiroptera	Rondinini, C., Di Marco, M., Chiozza, F., Santulli, G., Baisero, D., Visconti, P., Hoffmann, M., Schipper, J., Stuart, S.N., Tognelli, M.F., 2011. Global habitat suitability models of terrestrial mammals. Philos. Trans. R. Soc. B Biol. Sci. 366, 2633–2641.
Crocidura dolichura	Eulipotyphla	Rondinini, C., Di Marco, M., Chiozza, F., Santulli, G., Baisero, D., Visconti, P., Hoffmann, M., Schipper, J., Stuart, S.N., Tognelli, M.F., 2011. Global habitat suitability models of terrestrial mammals. Philos. Trans. R. Soc. B Biol. Sci. 366, 2633–2641.
Dendrohyrax arboreus	Hyracoidea	Rondinini, C., Di Marco, M., Chiozza, F., Santulli, G., Baisero, D., Visconti, P., Hoffmann, M., Schipper, J., Stuart, S.N., Tognelli, M.F., 2011. Global habitat suitability models of terrestrial mammals. Philos. Trans. R. Soc. B Biol. Sci. 366, 2633–2641.
Dendrohyrax dorsalis	Hyracoidea	Rondinini, C., Di Marco, M., Chiozza, F., Santulli, G., Baisero, D., Visconti, P., Hoffmann, M., Schipper, J., Stuart, S.N., Tognelli, M.F., 2011. Global habitat suitability models of terrestrial mammals. Philos. Trans. R. Soc. B Biol. Sci. 366, 2633–2641.
Rhynchocyon cirnei	Macroscelidea	Rondinini, C., Di Marco, M., Chiozza, F., Santulli, G., Baisero, D., Visconti, P., Hoffmann, M., Schipper, J., Stuart, S.N., Tognelli, M.F., 2011. Global habitat suitability models of terrestrial mammals. Philos. Trans. R. Soc. B Biol. Sci. 366, 2633–2641.
Smutsia gigantea	Pholidota	Rondinini, C., Di Marco, M., Chiozza, F., Santulli, G., Baisero, D., Visconti, P., Hoffmann, M., Schipper, J., Stuart, S.N., Tognelli, M.F., 2011. Global habitat suitability models of terrestrial mammals. Philos. Trans. R. Soc. B Biol. Sci. 366, 2633–2641.
Phataginus tetradactyla	Pholidota	Rondinini, C., Di Marco, M., Chiozza, F., Santulli, G., Baisero, D., Visconti, P., Hoffmann, M., Schipper, J., Stuart, S.N., Tognelli, M.F., 2011. Global habitat suitability models of terrestrial mammals. Philos. Trans. R. Soc. B Biol. Sci. 366, 2633–2641.

Phataginus tricuspis	Pholidota	Rondinini, C., Di Marco, M., Chiozza, F., Santulli, G., Baisero, D., Visconti, P., Hoffmann, M., Schipper, J., Stuart, S.N., Tognelli, M.F., 2011. Global habitat suitability models of terrestrial mammals. Philos. Trans. R. Soc. B Biol. Sci. 366, 2633–2641
Pan troglodytes	Primates	Plumptre, A.J., Nixon, S., Critchlow, R., Vieilledent, G., Kirkby, A., Williamson, E.A., Hall, J., Kujirakwinja, D., 2015. Status of Grauer's gorilla and chimpanzees in eastern Democratic Republic of Congo: Historical and current distribution and abundance.
Cercopithecus_denti	Primates	IUCN, 2020. The IUCN Red List of Threatened Species. http://www.iucnredlist.org
Perodicticus_ibeanus	Primates	IUCN, 2020. The IUCN Red List of Threatened Species. http://www.iucnredlist.org
Piliocolobus foai	Primates	IUCN, 2020. The IUCN Red List of Threatened Species. http://www.iucnredlist.org
Piliocolobus langi	Primates	IUCN, 2020. The IUCN Red List of Threatened Species. http://www.iucnredlist.org
Piliocolobus lulindicus	Primates	IUCN, 2020. The IUCN Red List of Threatened Species. http://www.iucnredlist.org
Piliocolobus semlikiensis	Primates	IUCN, 2020. The IUCN Red List of Threatened Species. http://www.iucnredlist.org
Gorilla beringei	Primates	Plumptre, A.J., Ayebare, S., Segan, D., Watson, J. & Kujirakwinja, D., 2016. Conservation Action Plan for the Albertine Rift. Rep. Wildl. Conserv. Soc. its Partners.
Cercopithecus ascanius	Primates	Rondinini, C., Di Marco, M., Chiozza, F., Santulli, G., Baisero, D., Visconti, P., Hoffmann, M., Schipper, J., Stuart, S.N., Tognelli, M.F., 2011. Global habitat suitability models of terrestrial mammals. Philos. Trans. R. Soc. B Biol. Sci. 366, 2633–2641.
Cercopithecus hamlyni	Primates	Rondinini, C., Di Marco, M., Chiozza, F., Santulli, G., Baisero, D., Visconti, P., Hoffmann, M., Schipper, J., Stuart, S.N., Tognelli, M.F., 2011. Global habitat suitability models of terrestrial mammals. Philos. Trans. R. Soc. B Biol. Sci. 366, 2633–2641.
Allochrocebus lhoesti	Primates	Rondinini, C., Di Marco, M., Chiozza, F., Santulli, G., Baisero, D., Visconti, P., Hoffmann, M., Schipper, J., Stuart, S.N., Tognelli, M.F., 2011. Global habitat suitability models of terrestrial mammals. Philos. Trans. R. Soc. B Biol. Sci. 366, 2633–2641.
Cercopithecus mitis	Primates	Rondinini, C., Di Marco, M., Chiozza, F., Santulli, G., Baisero, D., Visconti, P., Hoffmann, M., Schipper, J., Stuart, S.N., Tognelli, M.F., 2011. Global habitat suitability models of terrestrial mammals. Philos. Trans. R. Soc. B Biol. Sci. 366, 2633–2641.

Cercopithecus neglectus	Primates	Rondinini, C., Di Marco, M., Chiozza, F., Santulli, G., Baisero, D., Visconti, P., Hoffmann, M., Schipper, J., Stuart, S.N., Tognelli, M.F., 2011. Global habitat suitability models of terrestrial mammals. Philos. Trans. R. Soc. B Biol. Sci. 366, 2633–2641.
Colobus angolensis	Primates	Rondinini, C., Di Marco, M., Chiozza, F., Santulli, G., Baisero, D., Visconti, P., Hoffmann, M., Schipper, J., Stuart, S.N., Tognelli, M.F., 2011. Global habitat suitability models of terrestrial mammals. Philos. Trans. R. Soc. B Biol. Sci. 366, 2633–2641.
Colobus guereza	Primates	Rondinini, C., Di Marco, M., Chiozza, F., Santulli, G., Baisero, D., Visconti, P., Hoffmann, M., Schipper, J., Stuart, S.N., Tognelli, M.F., 2011. Global habitat suitability models of terrestrial mammals. Philos. Trans. R. Soc. B Biol. Sci. 366, 2633–2641.
Lophocebus albigena	Primates	Rondinini, C., Di Marco, M., Chiozza, F., Santulli, G., Baisero, D., Visconti, P., Hoffmann, M., Schipper, J., Stuart, S.N., Tognelli, M.F., 2011. Global habitat suitability models of terrestrial mammals. Philos. Trans. R. Soc. B Biol. Sci. 366, 2633–2641.
Papio anubis	Primates	Rondinini, C., Di Marco, M., Chiozza, F., Santulli, G., Baisero, D., Visconti, P., Hoffmann, M., Schipper, J., Stuart, S.N., Tognelli, M.F., 2011. Global habitat suitability models of terrestrial mammals. Philos. Trans. R. Soc. B Biol. Sci. 366, 2633–2641.
Chlorocebus tantalus	Primates	Rondinini, C., Di Marco, M., Chiozza, F., Santulli, G., Baisero, D., Visconti, P., Hoffmann, M., Schipper, J., Stuart, S.N., Tognelli, M.F., 2011. Global habitat suitability models of terrestrial mammals. Philos. Trans. R. Soc. B Biol. Sci. 366, 2633–2641.
Cercocebus agilis	Primates	Rondinini, C., Di Marco, M., Chiozza, F., Santulli, G., Baisero, D., Visconti, P., Hoffmann, M., Schipper, J., Stuart, S.N., Tognelli, M.F., 2011. Global habitat suitability models of terrestrial mammals. Philos. Trans. R. Soc. B Biol. Sci. 366, 2633–2641.
Loxodonta africana	Proboscidea	Plumptre, A.J., Ayebare, S., Segan, D., Watson, J. & Kujirakwinja, D., 2016. Conservation Action Plan for the Albertine Rift. Rep. Wildl. Conserv. Soc. its Partners.
Cricetomys ansorgei	Rodentia	IUCN, 2020. The IUCN Red List of Threatened Species. http://www.iucnredlist.org
Atherurus africanus	Rodentia	Rondinini, C., Di Marco, M., Chiozza, F., Santulli, G., Baisero, D., Visconti, P., Hoffmann, M., Schipper, J., Stuart, S.N., Tognelli, M.F., 2011. Global habitat suitability models

		of terrestrial mammals. Philos. Trans. R. Soc. B Biol. Sci. 366, 2633–2641.
Orycteropus afer	Tubulidentata	Rondinini, C., Di Marco, M., Chiozza, F., Santulli, G., Baisero, D., Visconti, P., Hoffmann, M., Schipper, J., Stuart, S.N., Tognelli, M.F., 2011. Global habitat suitability models of terrestrial mammals. Philos. Trans. R. Soc. B Biol. Sci. 366, 2633–2641.
Carbon	Ecosystem	Xu, L., Saatchi, S.S., Shapiro, A., Meyer, V., Ferraz, A., Yang, Y., Bastin, J.-F., Banks, N., Boeckx, P., Verbeeck, H., 2017. Spatial distribution of carbon stored in forests of the Democratic Republic of Congo. Sci. Rep. 7, 1–12.
Low dense rainforest south-eastern equatorial climate	Ecosystem	Shapiro, A.C., Grantham, H.S., Aguilar-Amuchastegui, N., Murray, N.J., Gond, V., Bonfils, D., Rickenbach, O., 2021. Forest condition in the Congo Basin for the assessment of ecosystem conservation status. Ecol. Indic. 122, 107268.
Sub dense rainforest south-eastern equatorial climate	Ecosystem	Shapiro, A.C., Grantham, H.S., Aguilar-Amuchastegui, N., Murray, N.J., Gond, V., Bonfils, D., Rickenbach, O., 2021. Forest condition in the Congo Basin for the assessment of ecosystem conservation status. Ecol. Indic. 122, 107268.
Montane dense rainforest south-eastern equatorial climate	Ecosystem	Shapiro, A.C., Grantham, H.S., Aguilar-Amuchastegui, N., Murray, N.J., Gond, V., Bonfils, D., Rickenbach, O., 2021. Forest condition in the Congo Basin for the assessment of ecosystem conservation status. Ecol. Indic. 122, 107268.
Low rainforest south-eastern equatorial climate	Ecosystem	Shapiro, A.C., Grantham, H.S., Aguilar-Amuchastegui, N., Murray, N.J., Gond, V., Bonfils, D., Rickenbach, O., 2021. Forest condition in the Congo Basin for the assessment of ecosystem conservation status. Ecol. Indic. 122, 107268.
Sub rainforest south-eastern equatorial climate	Ecosystem	Shapiro, A.C., Grantham, H.S., Aguilar-Amuchastegui, N., Murray, N.J., Gond, V., Bonfils, D., Rickenbach, O., 2021. Forest condition in the Congo Basin for the assessment of ecosystem conservation status. Ecol. Indic. 122, 107268.
Montane rainforest south-eastern equatorial climate	Ecosystem	Shapiro, A.C., Grantham, H.S., Aguilar-Amuchastegui, N., Murray, N.J., Gond, V., Bonfils, D., Rickenbach, O., 2021. Forest condition in the Congo Basin for the assessment of ecosystem conservation status. Ecol. Indic. 122, 107268.
Low Semi Rainforest northern equatorial climate	Ecosystem	Shapiro, A.C., Grantham, H.S., Aguilar-Amuchastegui, N., Murray, N.J., Gond, V., Bonfils, D., Rickenbach, O., 2021. Forest condition in the Congo Basin for the assessment of ecosystem conservation status. Ecol. Indic. 122, 107268.
Sub semi rainforest northern equatorial climate	Ecosystem	Shapiro, A.C., Grantham, H.S., Aguilar-Amuchastegui, N., Murray, N.J., Gond, V., Bonfils, D., Rickenbach, O., 2021. Forest condition in the Congo Basin for the assessment of ecosystem conservation status. Ecol. Indic. 122, 107268.

Montane Semi Rainforest northern equatorial climate	Ecosystem	Shapiro, A.C., Grantham, H.S., Aguilar-Amuchastegui, N., Murray, N.J., Gond, V., Bonfils, D., Rickenbach, O., 2021. Forest condition in the Congo Basin for the assessment of ecosystem conservation status. Ecol. Indic. 122, 107268.
Low Semi Rainforest southern equatorial climate	Ecosystem	Shapiro, A.C., Grantham, H.S., Aguilar-Amuchastegui, N., Murray, N.J., Gond, V., Bonfils, D., Rickenbach, O., 2021. Forest condition in the Congo Basin for the assessment of ecosystem conservation status. Ecol. Indic. 122, 107268.
Sub Montane Semi Rainforest southern equatorial climate	Ecosystem	Shapiro, A.C., Grantham, H.S., Aguilar-Amuchastegui, N., Murray, N.J., Gond, V., Bonfils, D., Rickenbach, O., 2021. Forest condition in the Congo Basin for the assessment of ecosystem conservation status. Ecol. Indic. 122, 107268.
Montane Semi Rainforest southern equatorial climate	Ecosystem	Shapiro, A.C., Grantham, H.S., Aguilar-Amuchastegui, N., Murray, N.J., Gond, V., Bonfils, D., Rickenbach, O., 2021. Forest condition in the Congo Basin for the assessment of ecosystem conservation status. Ecol. Indic. 122, 107268.
Low Semi Rainforest Pioneer southern equatorial climate	Ecosystem	Shapiro, A.C., Grantham, H.S., Aguilar-Amuchastegui, N., Murray, N.J., Gond, V., Bonfils, D., Rickenbach, O., 2021. Forest condition in the Congo Basin for the assessment of ecosystem conservation status. Ecol. Indic. 122, 107268.
Sub Montane Semi Rainforest	Ecosystem	Shapiro, A.C., Grantham, H.S., Aguilar-Amuchastegui, N., Murray, N.J., Gond, V., Bonfils, D., Rickenbach, O., 2021. Forest condition in the Congo Basin for the assessment of ecosystem conservation status. Ecol. Indic. 122, 107268.
Montane Semi Rainforest Pioneer southern equatorial climate	Ecosystem	Shapiro, A.C., Grantham, H.S., Aguilar-Amuchastegui, N., Murray, N.J., Gond, V., Bonfils, D., Rickenbach, O., 2021. Forest condition in the Congo Basin for the assessment of ecosystem conservation status. Ecol. Indic. 122, 107268.
North eastern low open forest northern equatorial climate	Ecosystem	Shapiro, A.C., Grantham, H.S., Aguilar-Amuchastegui, N., Murray, N.J., Gond, V., Bonfils, D., Rickenbach, O., 2021. Forest condition in the Congo Basin for the assessment of ecosystem conservation status. Ecol. Indic. 122, 107268.
North eastern sub montane open forest northern equatorial climate	Ecosystem	Shapiro, A.C., Grantham, H.S., Aguilar-Amuchastegui, N., Murray, N.J., Gond, V., Bonfils, D., Rickenbach, O., 2021. Forest condition in the Congo Basin for the assessment of ecosystem conservation status. Ecol. Indic. 122, 107268.
North eastern montane open forest northern equatorial climate	Ecosystem	Shapiro, A.C., Grantham, H.S., Aguilar-Amuchastegui, N., Murray, N.J., Gond, V., Bonfils, D., Rickenbach, O., 2021. Forest condition in the Congo Basin for the assessment of ecosystem conservation status. Ecol. Indic. 122, 107268.
Southern low open forest southern equatorial climate	Ecosystem	Shapiro, A.C., Grantham, H.S., Aguilar-Amuchastegui, N., Murray, N.J., Gond, V., Bonfils, D., Rickenbach, O., 2021. Forest condition in the Congo Basin for the assessment of ecosystem conservation status. Ecol. Indic. 122, 107268.
Southern sub montane open forest southern equatorial climate	Ecosystem	Shapiro, A.C., Grantham, H.S., Aguilar-Amuchastegui, N., Murray, N.J., Gond, V., Bonfils, D., Rickenbach, O., 2021. Forest condition in the Congo Basin for the assessment of ecosystem conservation status. Ecol. Indic. 122, 107268.

Appendix B – Maps of included threats

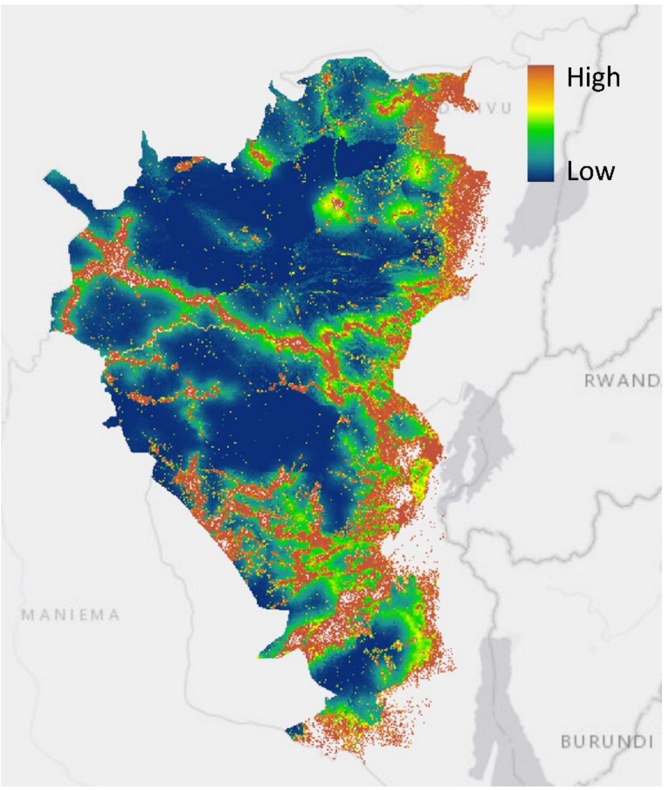

Figure A.B.1. Well-mapped threats represented by a forest intactness map (Grantham et al. 2020) which includes the current mappable threats of direct forest loss and fragmentation (remotely sensed), proximity to human settlements, population density, and accessibility, combined with a map depicting the results of a future deforestation risk model which is driven by predictable biophysical and anthropogenic factors of forest loss (Goldman et al. 2017).

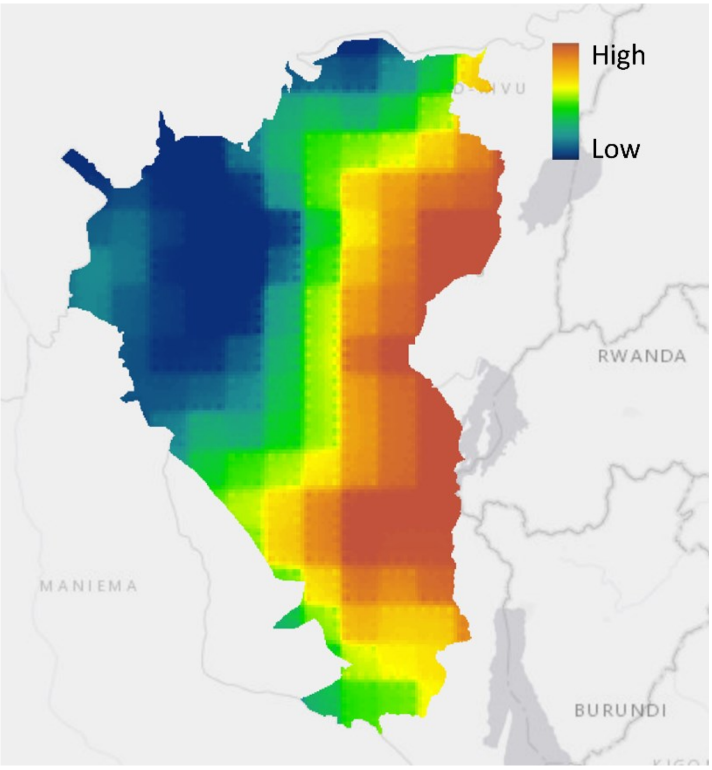

Figure A.B.2. The risk of armed conflict across the study region based on a combination of the probability of a conflict occurring, and the likely impact of that conflict (Hammill et al. 2016).

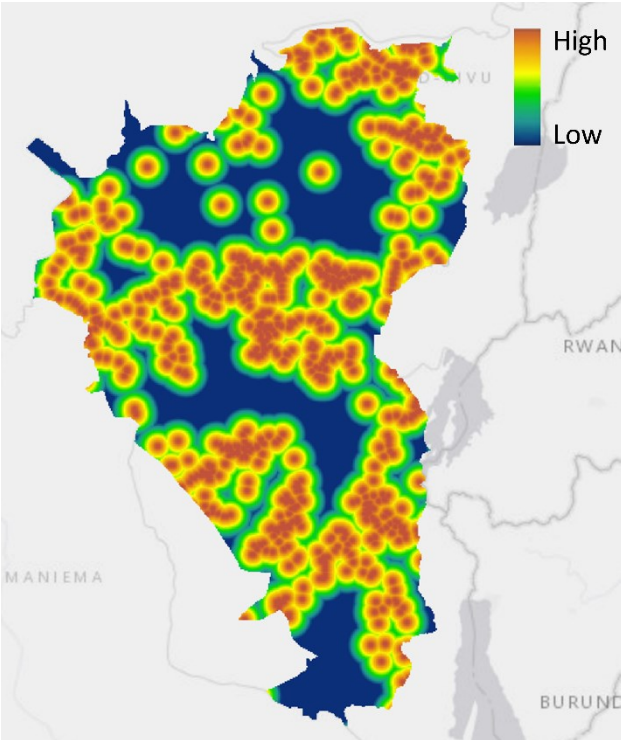

Figure A.B.3. The risk of potential ASM impacts based on a point information dataset for the eastern DRC (International Peace Information Service (IPIS) 2019) and a 20 km buffer around each mine location with impacts diminishing linearly (from 100% degraded at the point of impact to 0% degraded at 20 km distant from the point).

References

Goldman, E., N. Harris, and T. Maschler. 2017. *Predicting future forest loss in the Democratic Republic of Congo's CARPE Landscapes*.

Grantham, H. S., A. Duncan, T. D. Evans, K. R. Jones, H. L. Beyer, R. Schuster, J. Walston, J. C. Ray, et al. 2020. Anthropogenic modification of forests means only 40% of remaining forests have high ecosystem integrity. *Nature communications* 11. Nature Publishing Group: 1–10.

Hammill, E., A. I. T. Tulloch, H. P. Possingham, N. Strange, and K. A. Wilson. 2016. Factoring attitudes towards armed conflict risk into selection of protected areas for conservation. *Nature communications* 7.

International Peace Information Service (IPIS). 2019. DRC mining site data.
